# High‐Resolution Patterned Delivery of Chemical Signals From 3D‐Printed Picoliter Droplet Networks

**DOI:** 10.1002/adma.202412292

**Published:** 2025-04-30

**Authors:** Jorin Riexinger, Thomas Caganek, Xingzao Wang, Yutong Yin, Khoa Chung, Linna Zhou, Hagan Bayley, Ravinash Krishna Kumar

**Affiliations:** ^1^ Chemistry Research Laboratory Department of Chemistry University of Oxford 12 Mansfield Road Oxford OX1 3TA UK; ^2^ Medical Sciences Division University of Oxford Headley Way Oxford OX3 9DU UK; ^3^ Department of Engineering Science University of Oxford Parks Road Oxford OX1 3PJ UK; ^4^ Section of Structural and Synthetic Biology Department of Infectious Disease Imperial College London Sir Alexander Fleming Building, Imperial College Road London SW7 2AZ UK

**Keywords:** 3D printing, antimicrobial agent, droplet interface bilayers (DIBs), droplet network, gene expression, nanopore, patterning, synthetic tissue

## Abstract

Synthetic cells, such as giant unilamellar vesicles, can be engineered to detect and release chemical signals to control target cell behavior. However, control over the targeting of cell populations is limited due to poor spatial or temporal resolution and the inability of synthetic cells to deliver patterned signals. Here, 3D‐printed picoliter droplet networks are described that direct gene expression in underlying bacterial populations by patterned release of a chemical signal with temporal control. Shrinkage of the droplet networks prior to use achieves spatial control over gene expression with ≈50 µm resolution. Ways to store chemical signals in the droplet networks and to activate release at controlled points in time are also demonstrated. Finally, it is shown that the spatially‐controlled delivery system can regulate competition between bacteria by inducing the patterned expression of toxic bacteriocins. This system provides the groundwork for the use of picoliter droplet networks in fundamental biology and in medicine in applications that require the controlled formation of chemical gradients (i.e., for the purpose of local control of gene expression) within a target group of cells.

## Graphical abstract

3D‐printed picoliter droplet networks have been fabricated that control gene expression in bacterial populations by releasing chemical signals with precise spatial definition and high temporal resolution. This system of effector release is widely applicable, offering diverse applications in biology and medicine.

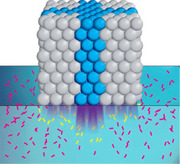

## 1 Introduction

The patterning of cells is crucial in nature, where it is required for the stability of bacterial communities and their species diversity,^[^ ^1^ ^]^ tissue development,^[^ ^2^ ^]^ morphogenesis^[^ ^3^ ^]^ and homeostasis,^[^ ^4^ ^]^ cell functions (such as stress responses),^[^ ^5^ ^]^ and the immune response.^[^ ^6^ ^]^ Patterning is mediated by gene expression, which is tightly regulated by intercellular communication both in space and time, ensuring that cells precisely coordinate their roles within a group. Intercellular communication is mediated by chemical signals, including quorum sensing molecules,^[^ ^7^ ^]^ growth factors,^[^ ^8^ ^]^ cytokines,^[^ ^8^ ^]^ hormones,^[^ ^9^ ^]^ and neurotransmitters.^[^ ^10^ ^]^

Current ways to study intercellular communication include microfluidics^[^ ^11^, ^12^ ^]^ and 3D printing,^[^ ^13^, ^14^, ^15^, ^16^, ^17^ ^]^ with which multiple cell types can be positioned accurately with respect to each other. Furthermore, spatially‐controlled gene expression has been achieved at the millimeter scale with chemical signals^[^ ^18^, ^19^, ^20^, ^21^ ^]^ and at the micrometer scale using light.^[^ ^22^, ^23^, ^24^, ^25^, ^26^ ^]^ However, external control over gene expression via chemical signals, which is the basis of diffusion‐mediated communication, has not been achieved at high spatial resolution. Therefore, a universal technology to both study and control patterned gene expression at micrometer resolution within a group of cells is desirable.

Meanwhile, a range of U.S. Food and Drug Administration (FDA)‐approved platforms exist for altering cellular behavior through the delivery of cargo (small molecules, drugs, peptide/proteins, DNA/RNA) via endocytic pathways such as cargo‐conjugation, and the encapsulation of cargo within lipid/polymer nanoparticles.^[^ ^27^, ^28^, ^29^ ^]^ Moreover, porous materials, such as metal–organic frameworks,^[^ ^30^ ^]^ covalent organic frameworks,^[^ ^31^, ^32^ ^]^ and zeolites^[^ ^33^ ^]^ are being developed as drug delivery platforms. Recently, synthetic cells^[^ ^34^, ^35^ ^]^ – micrometer‐sized systems that mimic aspects of cell function – have been used to alter cellular behavior in mycelial,^[^ ^36^ ^]^ bacterial,^[^ ^37^, ^38^, ^39^, ^40^, ^41^, ^42^, ^43^ ^]^ and eukaryotic cells^[^ ^44^, ^45^, ^46^, ^47^, ^48^ ^]^ through the triggered delivery of chemical signals that can change gene expression levels. However, the use of these systems to alter cellular behavior is limited due to their inability to release chemical signals in a patterned manner. Moreover, synthetic cells show poor storage capacities^[^ ^49^ ^]^ of chemical signals due to their limited volume.

Functional droplet networks,^[^ ^50^, ^51^, ^52^, ^53^, ^54^ ^]^ in other contexts also referred to as synthetic tissues;^[^ ^55^, ^56^, ^57^ ^]^ however, exhibit potential for releasing chemical signals with high spatial and temporal resolution, as they feature the patterning of compartments^[^ ^13^, ^15^, ^17^, ^58^ ^]^ and signaling between compartments both within a droplet network and with the immediate external environment at micrometer resolution.^[^ ^59^, ^60^ ^]^

Here, we present 3D‐printed picoliter droplet networks as a universal platform that can direct cellular activity by the patterned release of chemical signals. Specifically, we demonstrate the controlled release of chemical signals onto populations of homogeneously‐distributed *Escherichia coli* (*E. coli)* micro‐colonies and elicit patterned changes in gene expression by the precise tuning of cargo release dynamics. In addition, we developed a method to reliably shrink our droplet networks to achieve chemical signaling with micrometer resolution (≈50 µm). Our system shows improved cargo storage capacity compared to synthetic cell‐based systems, while retaining the ability to release chemical signals from a single compartment. Further, we show that our networks can be orientated in space through magnetism and that cargo release can be activated at defined times by the connection of reservoir networks. We illustrate the versatility of our system by the spatiotemporal control of the expression of colicin E7, a proteinaceous toxin produced by specific *E. coli* strains,^[^ ^61^ ^]^ hence directing the outcome of toxin‐driven bacterial competition. Taken together, our results pave the way towards applications of functional droplet networks in directing cellular patterning for applications in fundamental biology and medicine that require the local control of gene expression within a target group of cells.

## 2 Results and Discussion

### 2.1 Interfacing Bacterial Cells with Droplet Networks

**Figure 1:**
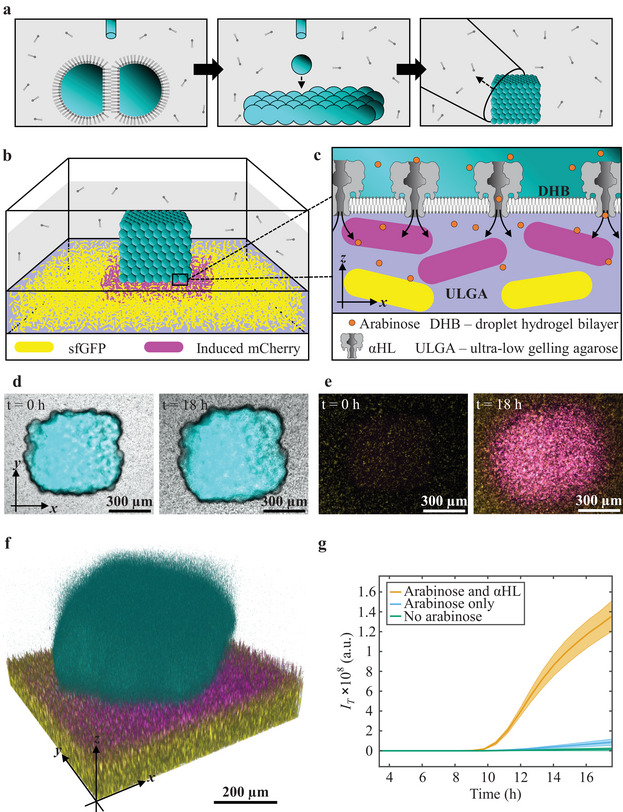
Localized gene expression. a) Schematic of the droplet printing process, where droplet interface bilayers (DIBs) are formed between the droplets (left image). Through 3D printing, networks of droplets are formed (center image), producing droplet networks, which can be transferred with a pipette (right image). b) A schematic of a 3D‐printed droplet network interfacing a bacterium‐laden hydrogel 18 h after transfer to the lipid‐in‐oil solution on top of the hydrogel. Magenta and yellow represent bacteria in the hydrogel expressing mCherry at high and low levels, respectively. c) Zoomed‐in schematic of (b) at the droplet network‐hydrogel interface. Droplet hydrogel bilayers (DHBs) form between the droplet network and the hydrogel. α‐Hemolysin (αHL) is incorporated into the bilayer, allowing arabinose to be released from the droplet network onto the immobilized bacteria, activating the expression of mCherry. d,e) Epi‐fluorescence microscopy images of a droplet network containing 33 × 10^−3^ м arabinose, 50 µg mL^−1^ αHL and 250 × 10^−6^ м cascade blue dextran on top of a bacterium‐laden hydrogel (composite bright‐field (d) and fluorescence images (e) at *t* = 0 h (when the droplet network is placed on top of the hydrogel) and *t* = 18 h (18 h after the droplet network is placed on top of the hydrogel)). f) A z‐stacked 3D confocal microscopy image of a droplet network on top of a bacterium‐laden hydrogel at 18 h. In (d)–(f), cyan is cascade blue dextran fluorescence, yellow represents sfGFP fluorescence and magenta represents mCherry fluorescence. g) A graph of the total mCherry expression, *I*_T_, overtime in bacterium‐laden hydrogels with droplet networks on top containing 33 × 10^−3^ м arabinose and 50 µg mL^−1^ αHL (orange line), only 33 × 10^−3^ м arabinose (blue line), or no arabinose (green line). Solid lines and shaded regions are the mean and standard deviation values of *n* = 3 technical repeats.

Patterned picoliter droplet networks – comprising 500–1000 droplets (≈150 pL per droplet) connected through droplet interface bilayers (DIBs) – were constructed by using a 3D droplet printer (**Figure** **1**a).^[^ ^56^ ^]^ In brief, aqueous droplets containing L‐(+)‐arabinose (the chemical signal used to induce bacterial protein expression) and monomers of the pore‐forming membrane protein α‐hemolysin (αHL) were ejected into a lipid‐in‐oil solution: 1,2‐diphytanoyl‐sn‐glycero‐phosphatidylcholine (DPhPC) and 1‐palmitoyl‐2‐oleoyl‐glycero‐3‐phosphocholine (POPC, 2:1 molar ratio) in 35:65 v:v undecane:silicone oil AR20). Monomers of αHL assemble to form heptameric transmembrane pores in the DIBs, which allow the diffusion of small molecules (less than 2 kDa)^[^ ^62^ ^]^ between the droplets. We have previously optimized the packing of our droplets within our 3D‐printed droplet networks to maximize hexagonal close‐packing.^[^ ^58^ ^]^

To interface these droplet networks with bacteria, droplet networks were transferred from the printing chamber (Figure 1a, right graphic) to a lipid‐in‐oil solution on top of a bacterium‐laden hydrogel (*E. coli* in 30 µL of 1.5% w/v ultra‐low gelling temperature agarose (ULGA), forming a cylindrically‐shaped hydrogel with a circular area of ≈0.32 cm^2^ and a thickness of ≈1 mm). *E. coli* cells were dispersed within the hydrogel at starting densities ranging from 1.6 × 10^7^ to 4.0 × 10^10^ cells mL^−1^. Once a droplet network (8 × 8 × 8 droplets) came into contact with the hydrogel, droplet hydrogel bilayers (DHBs) formed between the external droplets of the network and the lipid monolayer at the hydrogel surface that were stable for weeks, with an area of ≈0.3–0.4 mm^2^ (Figure 1b).^[^ ^63^ ^]^

To observe gene expression in the *E. coli* population (BZB1011 *Pmax:sfgfp::Tn7* pJS1‐*PBAD*:‐*mCherry*‐AMP – mCherry‐inducible (Table S1, Supporting Information)) by the release of arabinose from droplet networks, the bacteria contained the plasmid pJS1‐*PBAD*:‐*mCherry*‐AMP (Figure S1a, Supporting Information) encoding a fluorescent protein, mCherry, downstream of the promoter (P_BAD_), which is regulated by arabinose. Once droplet networks (initially containing 33 × 10^−3^ м arabinose and 25 µg mL^−1^ αHL monomer) were placed on top of the bacterium‐laden hydrogel (*t* = 0 h), αHL inserted into the DHBs, establishing a flux of arabinose through the pores into the bacterium‐laden hydrogel (Figure 1c). Over 18 h, we observed that *E. coli* cells were actively growing and dividing from single‐cell dispersions (*t* = 0 h, Figure 1d,e) to form 3D microcolonies,^[^ ^13^ ^]^ and cells directly below the droplet networks (within ≈50–100 µm from the contact area) and in close proximity to the droplet networks (within ≈100 µm from the edge) expressed high amounts of mCherry, quantified as the mean gene expression, *I*_M_ (see Experimental Section), of activated cells (634 a.u., Figure 1e,f). Cells further away from the droplet network expressed only baseline levels of mCherry (179 a.u., Figure 1e,f). An increase in fluorescence was detected at ≈*t* = 10 h (Figure 1g). This was followed by a rapid increase in mCherry expression, consistent with the all‐or‐nothing nature of the pBAD system (Note S1, Supporting Information). After ≈t = 18 h, no further increase in mCherry expression was detected. Droplet networks without αHL barely induced gene expression (Figure 1g), confirming the limited permeability of the lipid bilayers to arabinose,^[^ ^64^ ^]^ compared to other commonly used chemical signals, such as IPTG.^[^ ^65^ ^]^ Therefore, localized arabinose flux from droplet networks can be controlled by the permeabilization of bilayers with αHL pores, allowing localized gene expression within a homogenous population of bacterial cells.

### 2.2 Patterned Gene Expression Through Optimized Arabinose Flux

**Figure 2:**
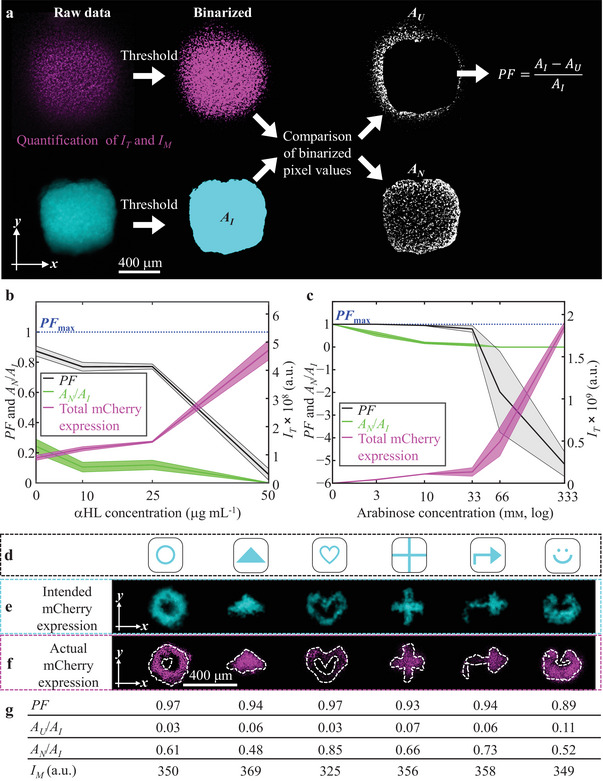
Patterned gene expression by controlled chemical signal release. a) Schematic of the image analysis workflow for calculating the pattern fidelity index (PF). First, raw epi‐fluorescence microscopy images of both mCherry expression and the droplet network containing cascade blue dextran are binarized, before comparing the binarized pixel values of the two epi‐fluorescence channels. Based on the area of intended gene expression (*A*_I_) the area of unintended expression (*A*_U_) and the area of no gene expression (*A*_N_) within *A*_I_ were computed. Then, PF as a measure of controlled release of the chemical signal was calculated by subtracting *A*_U_ from *A*_I_ before normalizing to A_I_ (see Equation (1) in text and Experimental Section). Total gene expression, *I*_T_, and mean gene expression, *I*_M_, were determined based on raw pixel values. b,c) Graphs of PF and total mCherry expression, *I*_T_ (see the Experimental Section), against αHL concentration (b) and arabinose concentration (c) within the droplets of the droplet networks. Solid lines and shaded regions, respectively, are the mean and standard deviation values of *n* = 4 technical repeats for droplet networks on top of bacterium‐laden hydrogels after 18 h. d) Schematic of cross‐sectional patterns incorporated into all layers of the 3D‐printed droplet networks. White represents droplets with no arabinose, no αHL, and no cascade blue dextran, and cyan respresents droplets containing 33 × 10^−3^ м arabinose, 25 µg mL^−1^ αHL and 250 × 10^−6^ м cascade blue dextran. e) Corresponding epi‐fluorescence images of droplet networks constructed according to the patterns in (d). Cyan fluorescence is cascade blue dextran. f) Binarized fluorescence images of the corresponding expression of mCherry (magenta) in bacteria underneath the droplet networks as shown in (e). e,f) Images 18 h after placement of the droplet networks on top of bacterium‐laden hydrogels. g) PF, unintended area of gene expression*(A*_U,_normalized to the intended area of gene expression, *A*_I_), area of no gene expression (*A*_N,_normalized to the intended area of gene expression, *A*_I_), and mean mCherry expression*(I*_M_ see the Experimental Section) corresponding to the patterns in (e),(f).

To understand and quantify the factors that control spatiotemporal release of arabinose and thus local gene expression within the bacterial population, we introduced the pattern fidelity (PF) index

(1) $$\mathrm{PF}=\frac{\left(A_{I}-A_{U}\right)}{A_{I}}\;$$

where *A*_I_ is the intended area of expression (the *x*, *y*‐plane cross‐sectional area of a droplet network where αHL inserts into the DHBs), and where *A*_U_ is the area of unintended expression (see the Experimental Section). We sought a PF approaching a value of 1, reflecting minimal gene expression in unintended areas, *A*_U_ (**Figure** **2**a), while maintaining a high level of local gene expression, by exploring the αHL and arabinose concentrations in the droplet networks. To evaluate control over gene expression within intended areas of gene expression, *A*_I_, we further introduced the normalized measure, *A*_N_, which indicated areas where no gene expression was induced within *A*_I_ (Figure 2a). At a fixed concentration of arabinose (33 × 10^−3^ м), we found that increased concentrations of αHL monomer (0–50 µg mL^−1^) led to decreased PF due to increasing *A*_U_, presumably caused by a greater flux of arabinose (Figure 2b). Moreover, we observed an increase in total gene expression, *I*_T_ (sum of all pixel values in activated pixels, see the Experimental Section), with increased αHL concentration (Figure 2b).We reasoned this was due to both increased gene expression in unintended areas of gene expression, *A*_U_, and increased gene expression within *A*_I_, which was confirmed by decreased areas of *A*_N_(area of no gene expression within *A*_I_). At a fixed concentration of αHL monomer (50 µg mL^−1^), arabinose concentrations higher than 33 × 10^−3^ м also led to decreased values of PF, associated with increasing *A*_U_ and *I*_T_ and decreasing areas of no gene expression within intended areas of gene expression, *A*_N_ (Figure 2c).

We found that at low αHL monomer (≤10 µg mL^−1^) and low arabinose (≤10 × 10^−3^ м) concentrations we could attain PFs close to 1; however, *I*_T_ was low (≤1.2 × 10^8^ a.u., Figure 2b,c). Therefore, we settled on concentrations of αHL monomer (25 µg mL^−1^) and arabinose (33 × 10^−3^ м) where PF was 0.77, but *I*_T_ was high (≥1.4 × 10^8^ a.u., Figure 2b,c) and *A*_N_ was low (0.12 ± 0.07, Figure 2b,c). At these concentrations, we printed a range of patterned droplet networks designed to produce different release patterns of chemical signals, by using droplets that did or did not contain arabinose and αHL. These printed networks produced gene expression patterns within a homogenous population of bacteria at PFs of ≥0.89 (Figure 2d–g; Note S2, Supporting Information). To further evaluate the control over induced gene expression as a consequence of arabinose release from 3D‐printed synthetic tissues, we quantified areas of no gene expression (*A*_N_) within areas of intended gene expression (*A*_I_). We observed *A*_N_ values between 0.48 and 0.85, which we reasoned were due to the fact that the hydrogels were not entirely flat and, therefore, some of the droplets did not form DHBs, preventing arabinose from being released into the bacterium‐laden hydrogel.

**Figure 3:**
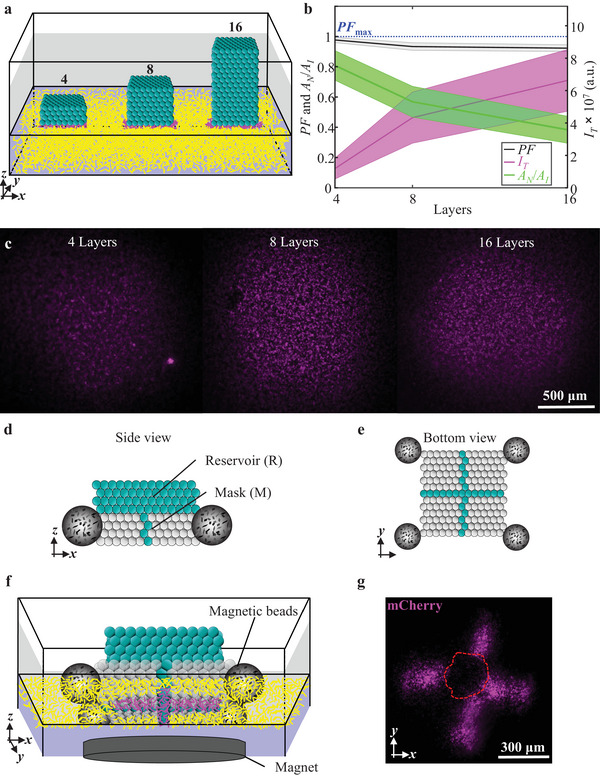
Chemical signal storage and release in droplet networks. a) Schematic of droplet networks of 4, 8, and 16 droplet layers on top of a bacterium‐laden hydrogel. b) Graph of the relationship between PF, total mCherry expression, *I*_T_, and normalized areas of no expression, *A*_N_, within *A*_I_ (normalized to *A*_I_) with increasing droplet layer numbers. Solid lines and shaded regions, respectively, are the mean and standard deviation values of *n* = 3 technical repeats for droplet networks on top of bacterium‐laden hydrogels after 18 h. c) Epi‐fluorescence images of mCherry at 18 h after transfer of droplet network networks composed of 4, 8, and 16 layers. d,e) Schematics depicting a droplet network composed of a mask (cross‐like pattern) with a reservoir on top (in cyan) from the side or bottom, respectively. Magnetic beads (grey spheres) are attached to the corners of the droplet network. f) Schematic of a droplet network composed of a mask and reservoir on top of a bacterium‐laden hydrogel after 18 h. A magnet (grey oval) directs controlled landing during the transfer of the droplet network to the bacterium‐laden hydrogel. g) Epi‐fluorescence image of mCherry at 18 h after droplet network transfer. The red dashed line indicates an area where the bottom droplets did not form the intended contact with the hydrogel. In the schematics (a) and (d)–(f) and the corresponding experiments in (b) and (g), cyan droplets contain 33 × 10^−3^ м arabinose, 50 µg mL^−1^ αHL and 250 × 10^−6^ м cascade blue dextran (see the Experimental Section), whereas white droplets in the schematics do not contain these three components. Magenta and yellow are representations of bacteria in the hydrogel expressing mCherry at high and low levels, respectively.

We next investigated whether we could store larger amounts of arabinose within printed droplet networks while maintaining spatial control over gene expression. Therefore, we sought to vary the number of layers (4, 8, 16 in total), while keeping *A*_I_ unchanged (**Figure** **3**a). The volume‐to‐surface‐area ratio (*R*_VSA_) was defined as

(2) $$R_{\mathrm{VSA}}=\frac{V}{A_{I}}\;$$

where *V* is the total volume of the droplet networks containing arabinose and αHL. According to this definition, droplet networks with an increasing number of network layers (4, 8, 16) are characterized by *R*_VSA_ values of 0.25, 0.49, and 1.07 mm, respectively. Increased *R*_VSA_ indeed led to enhanced total gene expression, *I*_T_, and decreased areas of *A*_N_ (with mean *A*_N_/*A*_I_ values of 0.81 ± 0.17, 0.57 ± 0.18, and 0.38 ± 0.15 for *R*_VSA_ values of 0.25, 0.49, and 1.07 mm, respectively, Figure 3b,c). Importantly, unintended expression, *A*_U_, increased only modestly (from *A*_U_/*A*_I_ values of 0.02 with 4‐layered networks to 0.08 with 16‐layered networks, corresponding to high PF values of 0.98 and 0.92, respectively, Figure 3b). To reduce printing time and further increase storage capacity (Note S3, Supporting Information), we printed droplet networks composed of 4 patterned layers at the bottom (mask) and 4 uniform layers at the top entirely composed of arabinose and αHL‐containing compartments (reservoir, Figure 3d,e). By equipping our droplet networks with magnetic handles (agarose droplets containing MagneHis Ni‐particles) and placing a magnet underneath the bacterium‐laden hydrogel, droplet networks were precisely guided to interface with the bacterium‐laden hydrogel through the mask layers rather than the reservoir layers (Figure 3f; Note S3 and Figures S2 and S3, Supporting Information), retaining patterned gene induction (Figure 3g).

### 2.3 Improved Resolution of Gene Expression with “Shrunken” Droplet Networks

**Figure 4:**
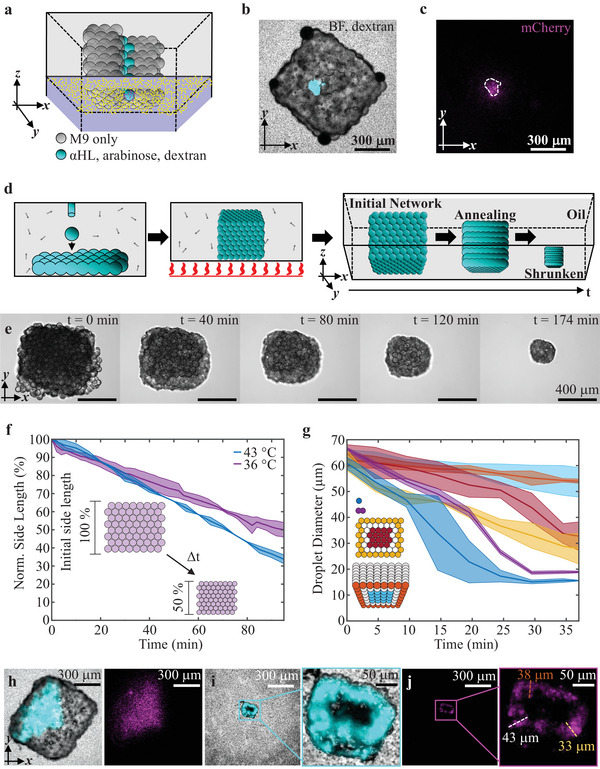
Optimized resolution of patterned gene expression. a–c) Schematic, composite (bright‐field (BF) and epi‐fluorescence (cascade blue dextran)) and epi‐fluorescence images, respectively, of a droplet network containing an αHL‐mediated diffusion pathway comprising a single linear chain of droplets after 18 h on top of a bacterium‐laden hydrogel. d) Schematic of a droplet network during the heat‐induced shrinking process, where a droplet network is printed in lipid‐in‐oil (left image), before placement on top of a heating plate (center image), which initiates the shrinking process through droplet annealing and water efflux (right image). e) Bright‐field microscopy images over time of a droplet network in lipid‐in‐oil solution on top of a heating plate set to 43 °C. f) Time‐dependence of the percentage changes in network side length (relative to initial side length) during heating at 36 and 43 °C (see the Experimental Section). g) Diameters over time of single droplets, droplets forming a single droplet interface bilayer with another droplet, and central and peripheral droplets of one‐layered droplet networks and eight‐layered droplet networks heated at 36 °C. In f,g) solid lines and shaded regions, respectively, are the mean values and standard deviations of *n* = 3 technical repeats. h–j) Composite (bright‐field and fluorescence) and epi‐fluorescence images of an unheated droplet network (triangular pattern, left) and a shrunken network (rectangular pattern, right) at 18 h after transfer on top of a bacterial‐laden hydrogel. The droplet network in i) was shrunken in lipid‐in‐oil solution for 170 min at 43 °C before transfer on top of the bacterium‐laden hydrogel. In (a)–(d) and (h)–(j) cyan droplets contain 33 × 10^−3^ м arabinose, 50 µg mL^−1^ αHL and cascade blue dextran, while white droplets do not contain these components. Magenta and yellow represent bacteria in the hydrogel expressing mCherry at high and low levels, respectively. In (j) the dashed lines represent the frame width of the frame‐like gene expression pattern.

We next sought to achieve even higher spatial resolution of patterned gene expression with droplet networks. We found that chemical signal release can be achieved through a linear, single‐droplet pathway (with droplet diameters of 65 µm) containing arabinose and αHL (**Figure** **4**a,b). In this case, gene expression was induced within a circular area of ≈100 µm diameter (Figure 4c). To further increase the resolution, we developed a heat‐induced postprinting shrinking process (Figure 4d). At the start of this process, we observed increasing contact angles between droplets, forming more tightly packed droplet networks (droplet annealing). Then, the volume of the compartments continuously decreased, presumably as water molecules partitioned into the oil, from which water molecules eventually were released to the unsaturated atmosphere in an evaporative process (Figure 4d). Importantly, the general morphology of the droplet networks (Figure 4e) and the patterned arrangements of the chemical signal‐containing droplets were barely affected (Figure 4i). We found that the rate at which the droplets shrank increased with temperature (Figure 4f), which we reasoned was due to the exponential increase in water vapor pressure^[^ ^66^ ^]^ and an increased solubility of water molecules in the oil phase with increasing temperature, causing water flow from droplet networks through the oil into the atmosphere. Additionally, we discovered that for a given droplet, the shrinking rate decreased with an increasing number of neighboring droplets. For example, we found that droplets at the periphery of droplet networks shrank more quickly than droplets in the center (Figure 4g), likely because water molecules from peripheral droplets partition into the oil phase directly. Solute concentrations could be diluted prior to printing, such that target concentrations were reached within the droplets after the evaporation process. By using shrunken droplet networks, we significantly increased spatial control compared to single‐droplet pathways (≈100 µm, Figure 4a) and previous patterns, such as a triangular pattern (Figure 4h). Shrunken networks activated gene expression within a frame‐like pattern, the width of which was ≤50 µm (Figure 4i,j), and therefore smaller than for single‐droplet pathways (Figure 4a).

### 2.4 Switchable Induction of Gene Expression

**Figure 5:**
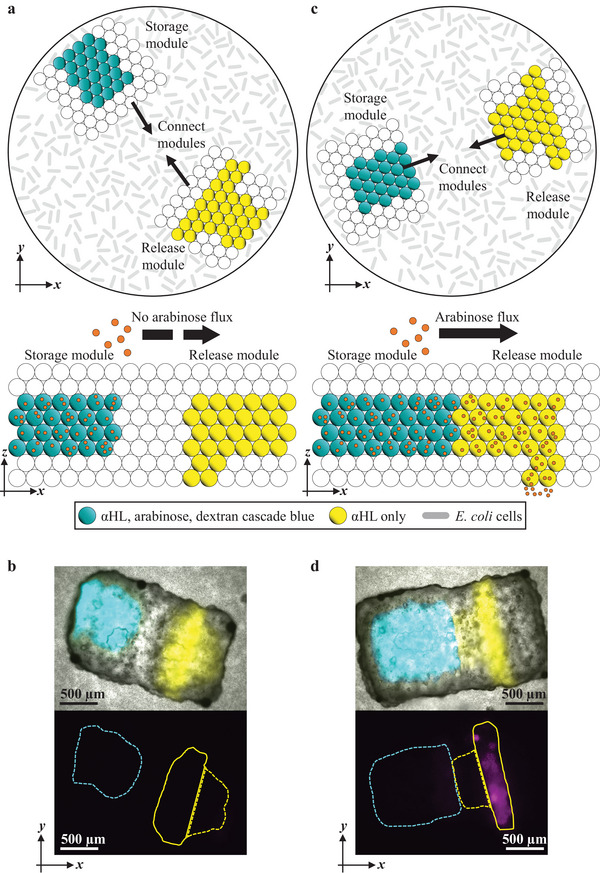
Droplet networks as switchable modules. a,c) Schematics of two droplet networks comprising droplets that contain cyan compartments: 33 × 10^−3^ м arabinose, 50 µg mL^−1^ αHL and 250 × 10^−6^ м cascade blue dextran; or yellow compartments: only 50 µg mL^−1^ αHL and 250 × 10^−3^ м cascade green dextran; or white compartments: neither αHL nor arabinose. The constructs are shown before and after establishing contact between the two modules. In (a) and (c), the modules are connected so that an αHL‐mediated diffusion pathway is either formed (c) or not formed (a). Orange circles represent arabinose. b,d) Composite (bright‐field and fluorescence) microscopy images and epi‐fluorescence images of connected droplet networks showing the corresponding gene expression patterns after 18 h where there is a diffusive pathway (d) or no diffusive pathway (b). In (b) and (d), cyan, yellow and magenta fluorescence are cascade blue dextran, cascade green dextran, and mCherry fluorescence, respectively. Dashed lines represent cross‐sectional outlines of droplets that contained αHL and arabinose (cyan) or only αHL (yellow). Yellow full lines represent cross‐sectional outlines of droplets that interfaced with the bacterium‐laden hydrogel and contained only αHL.

The release of arabinose is initiated once αHL pores insert into the DHBs formed between the droplet network and the bacterium‐laden hydrogel. We sought to activate the release by taking advantage of the modularity of droplet networks.^[^ ^67^ ^]^ We printed two separate droplet networks: one contained an arabinose reservoir (the storage module), and the other contained an αHL‐mediated droplet pathway (the release module). If the storage and release modules were not connected or connected incorrectly (**Figure** **5**a), no gene expression was induced (Figure 5b). In contrast, if the modules were assembled correctly, arabinose diffused into the bacterium‐laden hydrogel (Figure 5c), inducing patterned gene expression within the bacterial population (Figure 5d). By these means, we controlled the release of chemical signals by a key‐lock‐mechanism which couples two droplet network modules, allowing chemical signal release only once the two modules are connected correctly.

### 2.5 Patterned Bacterial Competition

Finally, to demonstrate the utility of patterned chemical signal release from droplet networks, the approach was applied to interference competition in bacterial communities.^[^ ^68^ ^]^ Specific *E. coli* strains can inhibit the growth of closely related strains by producing and releasing (by self‐lysis) proteinaceous toxins, such as DNA‐damaging colicins (e.g., colicin E7 and E8, Note S4.1–S4.6, Figures S4–S7, Supporting Information).^[^ ^61^, ^69^ ^]^

**Figure 6:**
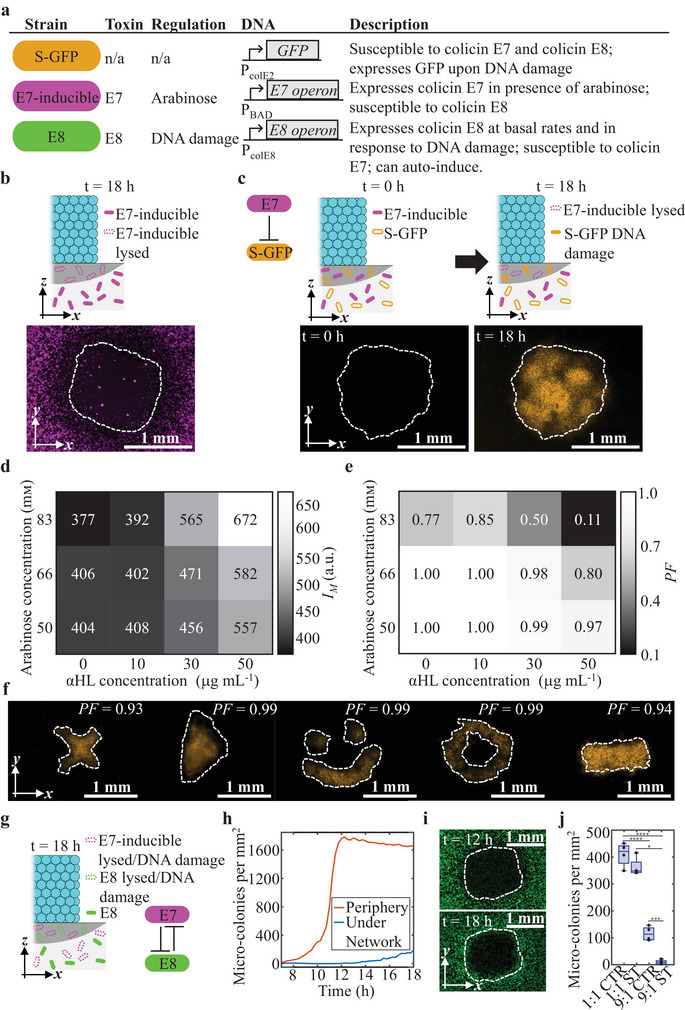
Patterned competition between bacteria. a) Overview of the competing strains: E7‐inducible (BZB1011 *Pmax:sfgfp::Tn7* pKC1‐*PBAD*:‐*ColE7*‐AMP, magenta) encoding the colicin E7 operon, and an mCherry reporter protein, inducible by arabinose; susceptible cells (BZB1011 pUA66‐*PcolE2:sfgfp*, orange) which are susceptible to colicin E7 and contain a plasmid encoding sfGFP downstream of the P_colE2_ promoter, which upregulates gene expression upon DNA damage; and E8 (BZB1011 *Pmax:mrfp1::Tn7* pColE8, green) which express colicin E8 at basal rates and regulate expression depending on DNA damage. b,c) Schematics and epi‐fluorescence images depicting local lysis of E7‐inducible (b) and local DNA damage in susceptible cells (c) upon release of chemical signal molecules from droplet networks. d,e) Heat maps of mean sfGFP expression, *I*_M_ (d), and pattern fidelity, PF (e), in S‐GFP cells as a consequence of arabinose release from droplet networks into a mixed population of E7R‐inducible (BZB1011 pYY1‐*PBAD*:‐*ColE7*‐*mCherry*‐AMP) and S‐GFP cells (1:1) at a range of arabinose and αHL concentrations after 18 h. f) Epi‐fluorescence microscopy images of bacterial populations containing E7R‐inducible and S‐GFP cells (1:1) under droplet networks that released arabinose in a patterned manner using PF‐maximizing arabinose (66 × 10^−3^ м) and αHL concentrations (30 µg mL^−1^) from (d) and (e) heatmaps. Images are at *t* = 18 h, orange reflects sfGFP fluorescence, and white dashed lines outline patterns. g) Schematic of the competition between E7‐inducible cells (BZB1011 *Pmax:sfgfp::Tn7* pKC1‐*PBAD*:‐*ColE7*‐AMP, magenta) and E8 cells. h) Graph of the number of micro‐colonies per mm^2^ below a droplet network releasing arabinose into a homogenous population of E7‐inducible and E8 cells (9:1 initial starting ratio) over time. i) Epi‐fluorescence images of bacterial competition between E7‐inducible (magenta) and E8 cells (green) representative for h) at 12 and 18 h. j) Boxplot of the number of E8 microcolonies in the center of the well with and without droplet networks (ST and CTR (control), respectively) at initial starting ratios of 1:1 and 9:1 between E7‐inducible and E8 cells. The individual data points are depicted for each condition with *n* = 4 technical replicates. It was tested whether the data was normally distributed using the Shapiro‐Wilk test (*p* < 0.05). If groups were normally distributed, significance between groups was tested performing a two‐sample *t*‐test. If data of at least one group was not normally distributed, significance between groups was tested by performing a Wilcoxon rank‐sum test. *p < 0.05, **p < 0.01, ***p < 0.001, and ****p < 0.0001.

To test the ability of droplet networks to spatially control competition between colicin‐producing cells and susceptible cells, we engineered an *E. coli* strain to produce colicin E7 and the associated immunity and lysis proteins in the presence of arabinose (BZB1011 *Pmax:sfgfp::Tn7* pKC1‐*PBAD*:‐*ColE7*‐AMP – E7‐inducible, **Figure** **6**a; Table S1, Figure S1b, Supporting Information). When droplet networks containing arabinose (333 × 10^−3^ м) and αHL monomer (50 µg mL^−1^) were placed on top of bacterium‐laden hydrogels containing inducible E7 cells, the number of micro‐colonies underneath the droplet networks was significantly reduced from 927 ± 74 to 62 ± 42, indicating colicin E7 release from the inducible cells through cell lysis (Figure 6b; Figure S8a,b, Note S4.7, Supporting Information).

Next, we used a reporter plasmid that produces sfGFP upon DNA damage (BZB1011 pUA66‐*PcolE2:sfgfp* – S‐GFP, Figure 6a; Table S1, Note S4.8, Supporting Information)^[^ ^69^, ^70^ ^]^ to investigate DNA damage in S‐GFP cells as a consequence of colicin E7 exposure. For this, E7R‐inducible (BZB1011 pYY1‐*PBAD*:‐*ColE7*‐*mCherry*‐AMP – E7R‐inducible, Table S1, Figure S1c, Supporting Information) and S‐GFP cells were mixed homogenously within the hydrogel at an initial ratio of 1:1 (Figure 6c). In the absence of arabinose, E7R‐inducible and S‐GFP cells grew at similar rates with negligible DNA damage in the S‐GFP cells (as observed by the lack of sfGFP expression) over 18 h (Note S4.8, Figure S9a,b, Supporting Information). By releasing arabinose from the droplet networks (50 × 10^−3^ м arabinose, 50 µg mL^−1^ αHL monomer) into bacterium‐laden hydrogels containing E7R‐inducible and S‐GFP cells at an initial ratio of 1:1, local DNA damage in S‐GFP was observed underneath the droplet networks (Figure 6c; Figure S9c, Supporting Information), which was confirmed to be correlated with cell growth inhibition (Note S4.8; Figure S9b, Supporting Information).

To maximize the mean sfGFP expression (DNA damage) and the PF of DNA damage, droplet networks containing ranges of αHL and arabinose concentrations were placed on top of bacterium‐laden hydrogels containing both E7R‐inducible and S‐GFP cells. We found that increased arabinose and αHL concentrations in droplet networks led to increased DNA damage in S‐GFP cells (Figure 6d; Figure S9c and S10, Supporting Information) but decreasing PF values (Figure 6e). To achieve patterned DNA damage in S‐GFP cells as a result of colicin E7 expression, droplet networks containing 66 × 10^−3^ м arabinose and 30 µg mL^−1^ αHL were printed, as this combination produced an intermediate level of DNA damage (mean sfGFP expression (*I*_M_) of 471 a.u.), at a high PF value of 0.98. By using these conditions, patterned DNA damage was achieved with different printed patterns at PFs between 0.93 and 0.99 (Figure 6f, Supporting Information).

Finally, we investigated control of the competition between E7‐inducible and the natural colicin E8‐expressing strain (BZB1011 *Pmax:mrfp1::Tn7* pColE8 – E8, Figure 6g; Table S1, Supporting Information). When droplet networks induced expression of colicin E7 through arabinose release (at 333 × 10^−3^ м arabinose and 50 µg mL^−1^ αHL), the number of E8 cells was significantly reduced within a localized area underneath the network. We hypothesized that the elimination after 12 h (Figure 6h,i) was caused by high local concentrations of colicin E7 through lysis, inducing E8 to counter‐attack (as the natural E8 operon is regulated by DNA damage) through self‐lysis (Note S4.1, Supporting Information). We found that levels of colicin E7 expression and release from E7‐inducible cells necessary to eliminate colicin E8‐expressing cells were only reached at an initial starting ratio, the ratio between cells of one over the other genotype, of 9:1 (Figure 6j, see Experimental Section (Definitions and Calculations)). Interestingly after 18 h, E8 cells were able to partially grow back into the cleared area (Figure 6i). In summary, we have shown that droplet networks can locally control which strains win or lose in well‐mixed bacterial communities through the patterned‐release of chemical signals that upregulate toxin production in bacterial strains.

## 3 Conclusion

Here, we present chemical signal‐based communication between 3D‐printed droplet networks and bacterial cells. The flux of arabinose, the chemical signal, can be precisely controlled, by adjusting signal and pore concentrations within the droplet networks, to produce patterned gene expression in bacterial populations. We can magnetically guide our droplet networks to land precisely onto bacterial populations, controlling where chemical signal molecules can be released. In addition, we can store chemical signals in printed droplet reservoirs, while maintaining high spatial control over gene expression. We further increased the resolution of patterned gene expression significantly to 50 µm by using evaporative shrinkage of the networks, which represents a significant improvement when compared with the millimeter resolution of previous work.^[^ ^18^, ^19^, ^20^, ^21^, ^23^, ^71^ ^]^ Further, by using our key‐lock mechanism the release of chemical signals can be activated at a controlled point in time.

Critically, we show the utility of our system by inducing the expression of bacteriocins in *E. coli*, which drives patterned DNA damage in cocultured susceptible cells and local competition between toxin‐producing strains with different potencies. Therefore, the present work demonstrates chemical signal release from 3D‐printed droplet networks as a tool for the study of spatiotemporal dynamics of complex cellular behaviors within microbial communities.

Additionally, we envision that our droplet networks might encapsulate cell‐free protein expression systems,^[^ ^72^ ^]^ which could mediate the release of chemical signals in response to the environment. For example, *n*‐acyl homoserine lactones^[^ ^46^ ^]^ released from bacterial cells could drive the expression of αHL inside droplet networks, which would then allow chemical signals, such as arabinose, to be released back onto the bacterial cells, inducing the expression of a gene of interest, thereby, establishing bidirectional communication between droplet networks and bacterial cells. To further control chemical signal release, membrane proteins that gate in response to stimuli, such as Zn^2+[^ ^67^, ^73^, ^74^ ^]^ or light,^[^ ^75^ ^]^ could be used to permeabilize membranes to achieve light‐sensitive chemical signal release.^[^ ^75^ ^]^

We envisage that our modular system can encapsulate different types of chemical signals (such as drugs, peptide/proteins, or DNA/RNA), as well as having the ability to integrate with other effector technologies (for example lipid nanoparticles) for interactions with various cell types. Membrane pores with larger internal diameters could be used to allow larger signaling molecules to be released from droplet networks.^[^ ^46^ ^]^ For example, perfringolysin O has a diameter between 25 and 30 nm^[^ ^76^ ^]^ as compared with the αHL pore, whose narrowest inner diameter is 1.4 nm.^[^ ^62^ ^]^ Moreover, chemical signals could be produced in droplet networks by cell‐free protein expression or multiple types of droplet network could be connected to restore chemical signals once depleted (e.g., by exchanging storage modules) and to release multiple types of chemical signals (e.g., by connecting storage modules containing different chemical signals to a release module).

Further, our droplet networks could function in bulk aqueous environments by using external lipid bilayers surrounding the droplet networks to interface with cells in 3D.^[^ ^59^ ^]^

Lastly, 3D‐printed hybrid tissues that contain living cells might be constructed,^[^ ^13^, ^15^, ^17^ ^]^ where gradients of growth factors from droplet networks control the differentiation of cells at defined locations, thereby allowing the formation of complex tissues.^[^ ^77^, ^78^, ^79^ ^]^

In summary, our technology demonstrates patterned spatiotemporal communication between picoliter droplet networks and living cells based on chemical signals. The platform might be modified to investigate the patterning of various groups of cells through the release of various natural and synthetic signals, such as quorum sensing molecules, antibiotics and growth factors. Such an approach will prove useful in fundamental research, including the modulation of species diversity in bacterial communities or the spatiotemporal dynamics of tissue development and morphogenesis. Furthermore, we envision the use of droplet networks in medical applications,^[^ ^80^, ^81^, ^82^, ^83^ ^]^ such as in the treatment of chronic wounds, cancer, neurodegenerative diseases, and spinal cord injuries.

## 4 Experimental Section

### Preparing Aqueous Phases

Lysogeny broth Miller (LB) medium (Invitrogen) was prepared by adding LB powder (25 g) to Milli‐Q water (1 L) and autoclaving. 5 × M9 minimal salts solution was prepared by adding M9 minimal salts (2.82 g, Sigma‐Aldrich) to Milli‐Q water (100 mL) and autoclaving. Casamino acid solution was prepared by adding Calbiochem OmniPur Casamino acid (5 g, Merck) to Milli‐Q water (50 mL) and autoclaving. The final M9 minimal medium consisted of 1 × M9 minimal salts, MgSO_4_ (2 × 10^−3^ м), CaCl_2_ (0.1 × 10^−3^ м, filter‐sterilized using a 0.22‐µm polyethersulfone membrane (Millex‐GP Syringe Filter Unit)), casamino acids (0.2% w/v, Sigma) and d‐glucose (24 × 10^−3^ м) or glycerol (24 × 10^−3^ м). Bacterium‐laden hydrogels were prepared by adding ultralow gelling agarose (Sigma‐Aldrich) to the M9 minimal medium (at a final concentration of 1.5% w/v) and autoclaving. The hydrogel solution was kept molten in a water bath at 37 °C and prepared freshly before every experiment. Antibiotics were dissolved in Milli‐Q water, filter‐sterilized (0.22‐µm polyethersulfone membrane) and frozen (−20 °C) as stock solutions (ampicillin: 100 mg mL^−1^, kanamycin: 50 mg mL^−1^, Sigma‐Aldrich). The final concentrations of antibiotics in media were 100 and 50 µg mL^−1^ for ampicillin and kanamycin, respectively.

### Construction of Recombinant DNA

The *E. coli* strain BZB1011 was used for all Golden Gate cloning experiments in this study. *E. coli* cells were cultured in LB medium at 37 °C with shaking at 250 rpm and plated on LB agar, incubated at 37 °C. The LB medium and agar were supplemented with the appropriate antibiotics, either ampicillin (100 µg mL^−1^) or kanamycin (50 µg mL^−1^). For blue‐white screening, agar plates were additionally supplemented with IPTG (0.1 × 10^−3^ м, Sigma Aldrich) and Xgal (40 µg mL^−1^, Thermo Fisher). Constructs (pKC1‐*PBAD*:‐*ColE7*‐AMP and pYY1‐*PBAD*:‐*ColE7*‐*mCherry*‐AMP, see Table S1 in the Supporting Information) were made using the MoClo kit and cloning method.^[^ ^84^ ^]^ The following components were added to a 0.2 mL PCR tube: DNA components (10 fmol each), BsaI (10 U) or BbsI (10 U) restriction enzyme (NEB), T4 Ligase (20 U, NEB), and 1 × was then added to bring the total volume to 10 µL. Reaction mixtures were incubated in a thermocycler for 40 cycles of digestion and ligation (37 °C for digestion for 2 min, 16 °C for ligation for 5 min), followed by 5 min at 50 °C and a heat kill step at 80 °C for 10 min. The mixtures were then held at 4 °C, and 4 µL was used to transform cells. Transformants were selected using lacZα blue‐white screening.

### Competent Cell Preparation and Cell Transformation

An overnight culture of *E. coli* BZB1011 cells was set up by inoculating the cells into LB medium, followed by shaking (225 rpm) at 37 °C for no longer than 12 h. The overnight culture was inoculated into LB (50 mL) at an OD_600_ of 0.05 and grown at 37 °C with shaking (225 rpm) until the OD_600_ reached 0.4‐0.6. The culture was centrifuged at 4481 × g at 4 °C for 10 min. The supernatant was removed and the cell pellet was resuspended in a solution (25 mL) containing CaCl_2_ (100 × 10^−3^ м) and glycerol (15% v/v) at 4 °C. After 45 min, the cells were pelleted again by centrifugation at 4481 × g at 4 °C for 10 min. The supernatant was removed and the cell pellet was resuspended in a solution (5 mL) containing CaCl_2_ (100 × 10^−3^ м) and glycerol (15% v/v) at 4 °C before aliquoting and storing at −80 °C. Chemically competent *E. coli* cells (BZB1011, 750 µL) were thawed on ice and the plasmid of interest (100 ng) was added. After 30 min on ice, the cells were heat‐shocked for 45 s at 42 °C. After 2 min on ice, the cells were added to prewarmed super optimal broth (SOC, 1 mL) at 37 °C and incubated for 1 h. The cells were then inoculated onto LB‐agar plates supplemented with antibiotics (100 µg mL^−1^ ampicillin, 50 µg mL^−1^ kanamycin). After overnight culture at 37 °C, a single colony was picked and inoculated into LB (4 mL), supplemented with antibiotics (100 µg mL^−1^ ampicillin, 50 µg mL^−1^ kanamycin), and shaken for no longer than 12 h at 37 °C (225 rpm). The culture was then mixed with an equal volume of glycerol (50% v/v) solution. Glycerol stocks were stored at −80 °C.

### Preparation of Bacterium‐Laden Hydrogels

*E. coli* cells (BZB1011) were pipetted from glycerol stocks into a round bottom tube containing LB (4 mL) supplemented with antibiotics (100 µg mL^−1^ ampicillin, 50 µg mL^−1^ kanamycin). Cells were grown for no longer than 12 h at 37 °C with shaking (225 rpm). 40 µL of overnight culture were then transferred to a round bottom tube containing LB (4 mL) supplemented with antibiotics (100 µg mL^−1^ ampicillin, 50 µg mL^−1^ kanamycin) and grown for 3 h at 37 °C with shaking (225 rpm). Based on the OD_600_, which was measured using a spectrophotometer (BioRad SmartSpec Plus, with a conversion factor of 1.0 = 10^8^ cells mL^−1^), appropriate amounts of culture were added to a 1.5 mL tube and centrifuged for 8 min at 8000 × g. The supernatant was removed and the cells were resuspended in appropriate amounts of preheated (37 °C) M9 medium containing ultralow gelling agarose (final concentration: 1.5% w/v) to form a molten bacterium‐laden hydrogel solution with the desired cell density (i.e., OD_600_ 0.02, 2, or 4.5). In case of experiments involving two bacterial strains (i.e., bacterial interference competition) the amount of cells were adjusted according to the starting ratio to reach the desired total cell concentration. The resulting cell suspension (30 µL) was added to a number of wells (<30) of a 96‐well plate according to the number of experimental conditions and solidified at 4 °C for 35 min.

### Expression and Purification of αHL

The αHL monomers were prepared by transforming *E. coli* BL21(DE3) pLysS cells (Agilent) with the pT7‐αHL‐D_8_H_6_ plasmid^[^ ^85^ ^]^ and inoculated onto LB‐agar plates containing antibiotics (carbenicillin, 50 µg mL^−1^, chloramphenicol, 34 µg mL^−1^). A single colony from the plate was picked and inoculated in LB (10 mL) for the preculture. An LB culture (400 mL) containing the same antibiotics was inoculated with overnight preculture (4 mL). This expression culture was shaken at 37 °C at 250 rpm for ≈3 h until the OD_600_ reached 0.6, when it was cooled to 18 °C before the addition of IPTG (2 mL, 0.1 м, Fluorochem) to induce protein expression. The culture was further shaken at 18 °C at 200 rpm overnight. The cells were then harvested by centrifugation in a Beckman J25 centrifuge at 5000 rpm for 20 min at 4 °C and resuspended in lysis buffer (10 mL, 50 × 10^−3^ м Tris‐HCl, pH 8.0, 150 × 10^−3^ м NaCl, 10 × 10^−3^ м imidazole, 0.1% Triton X‐100, 5% glycerol, 2 × 10^−3^ м TCEP with an EDTA‐free protease‐inhibitor tablet (ThermoFisher)). Lysis was then performed by the addition of lysozyme (250 µL, 40 mg mL^−1^, ThermoFisher), universal nuclease (2 µL, 250 U µL^−1^, ThermoFisher) and MgCl_2_‐containing solution (25 µL, 2 м), and incubation on ice for 1 h. The lysate was sonicated at 40% amplitude for 3 min in a 30 s‐ON‐30 s‐OFF pulse train on ice (VCX 500, Sonics). The supernatant was cleared by centrifugation at 29 000 × g for 45 min at 4 °C and transferred to a gravity column containing Ni‐NTA resin (1 mL, bed volume, ThermoFisher). The lysate supernatant and resin mixture were mixed at 4 °C on a platform rotator for 1 h. The column was washed with washing buffer (2 × 15 mL, 50 × 10^−3^ м Tris‐HCl, pH 8.0, 500 × 10^−3^ м NaCl, 20 × 10^−3^ м imidazole, 2 × 10^−3^ м TCEP, 0.1% Triton X‐100, and 5% glycerol) and eluted with elution buffer (50 × 10^−3^ м Tris‐HCl, pH 8.0, 500 × 10^−3^ м NaCl, 250 × 10^−3^ м imidazole, 2 × 10^−3^ м TCEP, 0.1% Triton X‐100 and 5% glycerol). The fractions (≈10 mL) containing αHL were combined and loaded onto a HiLoad 26/600 Superdex 200pg (Cytiva) SEC column equilibrated with SEC buffer (10 × 10^−3^ м Tris‐HCl, pH 8.0, 150 × 10^−3^ м NaCl, 2 × 10^−3^ м TCEP, and 5% glycerol) at 4 °C. Fractions containing monomers of αHL were concentrated (1 mg mL^−1^) and stored at −80 °C as aliquots. The mass of the monomer was verified by LC‐MS. On average, 9 mg pure αHL monomer after SEC purification was obtained from culture (400 mL).

### Preparation of Lipid‐in‐Oil Solutions

Lipids (1,2‐diphytanoyl‐sn‐glycero‐3‐phosphocholine (DPhPC, 4ME 16:0‐18:1 PC), 1‐palmitoyl‐2‐oleoyl‐glycero‐3‐phosphocholine (POPC, 16:0‐18:1 PC); Avanti Polar Lipids) were dissolved in anhydrous chloroform (2.5 mL, 10 mg mL^−1^, Sigma‐Aldrich). The final lipid composition DPhPC:POPC (2: 1 molar ratio) was prepared in chloroform‐cleaned, Teflon capped glass vials (Supelco). The chloroform was evaporated under nitrogen and the remaining solvent removed by placing the vials under vacuum for 24 h. The vials were stored at −80 °C under argon. Before use, the vials were brought to room temperature for 15 min and a premixed oil solution consisting of undecane (Sigma‐Aldrich) and silicone oil (AR20, Wacker) in a ratio of 35:65 v/v was added. The lipid‐in‐oil solution was vortexed and then sonicated (Branson 2800 ultrasonic bath 230 V) for 1 h at 25–35 °C and vortexed again before use. The total concentration of lipids was 2 × 10^−3^ м.

### 3D‐Printing of Droplet Networks

The droplet networks used in this work were formed by using a 3D‐printing device as described elsewhere.^[^ ^60^ ^]^ Briefly, an aqueous solution (M9 minimal medium supplemented with various concentrations of L‐(+)‐arabinose (0–333 × 10^−3^ м, Sigma‐Aldrich), αHL monomer (0–50 µg mL^−1^), and cascade blue dextran (250 × 10^−6^ м, Invitrogen, Cat. D1976) was ejected from a glass nozzle into the lipid‐in‐oil solution in a printing cuvette. The printing cuvettes (composed of special optical glass (SOG), Starna Scientific) were mounted on a micromanipulator stage (Patch Star 7000, Scientifica), which moved in *xyz*‐direction so to position the static glass nozzle according to a printing map. During droplet ejection, monolayers of lipid assemble spontaneously at the interface between the aqueous droplets and the lipid‐in‐oil solution. Lipid bilayers form between neighboring droplets when lipid monolayers contact one another. The placement of individual droplets with various contents can be controlled by using a multiple nozzle setup, where patterned droplet networks are formed by initializing the printing software according to the relative position of the glass nozzles. The droplet size (60–120 µm in diameter) was controlled by adjusting pulse voltage and/or pulse duration of the piezo driver.

### Placement of Droplet Networks on Bacterium‐Laden Hydrogels

Lipid‐in‐oil solution (50 µL) was pipetted on top of bacterium‐laden hydrogels in a 96‐well plate and incubated at room temperature for 15 min. Then, droplet networks were transferred to the lipid‐in‐oil solution using a pipette, when they sank forming robust DHBs at the interface with the hydrogel. After network placement the 96‐well plates were incubated at 37 °C for 18 h until further analysis (see pattern fidelity quantification by image analysis).

### Pattern Fidelity (PF) Quantification by Image Analysis

Bacterium‐laden hydrogels and droplet networks were imaged with an epi‐fluorescence microscope (Leica DMI8, camera: DFC7000T) and a laser scanning confocal microscope (Zeiss LSM780). Epi‐fluorescence images were recorded after placement of the droplet networks (at *t* = 0 h and *t* = 18 h, unless stated otherwise). The same settings were used throughout this work with 5 × magnification (objective: N PLAN 5x/0.12 DRY): cascade blue dextran: *λ*_ex_: 327–383 nm, *λ*_em_: 435–485 nm, exposure time: 600 ms, gain: 1; sfGFP: *λ*_ex_: 450–490 nm, *λ*_em_: 500–550 nm, exposure time: 200 ms (for constitutive expression of sfGFP) or 1 s (for bacterial interference competition), gain: 1; mCherry: *λ*_ex_: 540–552 nm, *λ*_em_: 567–643 nm, exposure time: 2 s, gain: 1. Images were composed of 1920 pixels in *x*‐direction (*x*_max_) and 1440 pixels in *y*‐direction (*y*_max_), spanning a field of view of 2.4887 mm (*x*‐direction) by 1.8662 mm (*y*‐direction) and were analyzed by using MATLAB R2021b. The pixel value range spanned 0–4095. Baseline gene expression of mCherry, *I*_B_, arising from bacterium‐laden hydrogels without arabinose, was computed based on the mean of all pixel intensity values $I_{x,y_{\mathrm{mCherry}}}$ in fluorescence images, where *x* is the *x* ^th^ pixel in *x*‐direction and *y* is the *y* ^th^ pixel in *y*‐direction of an image)

(3) $$I_{B}=\frac{\sum_{x=1}^{x_{\max}}\sum_{y=1}^{y_{\max}}I_{x,y_{\mathrm{mCherry}}}}{x_{\max}y_{\max}}=179\;a.u.$$

Accordingly, pixel intensity values ≥179 a.u. were considered to reflect induced gene expression of mCherry (termed activated pixels). The mean gene expression, *I*_M_, of all activated pixels in mCherry fluorescence images was computed as follows

(4) $$I_{M}=\frac{\sum_{x=1}^{x_{\max}}\sum_{y=1}^{y_{\max}}I_{x,y_{\mathrm{mCherry}}}\;\times \;H\left(I_{x,y_{\mathrm{mCherry}}}-I_{B}\right)}{N_{a}}\;$$

where *H*(*z*) is the Heaviside step function, defined as:

(5) $$H\;\left(z\right)=\left\{\begin{array}{c} 1\;\mathrm{if}\;z\geq 0\\ 0\;\mathrm{if}\;z<0 \end{array}\right.\;$$

and where *N*_a_ is the total number of activated pixels.

The total gene expression, *I*_T_, was calculated as the sum of pixel values of all activated pixels in mCherry fluorescence images

(6) $$I_{T}=\sum_{x=1}^{x_{\max}}\sum_{y=1}^{y_{\max}}I_{x,y_{\mathrm{mCherry}}}\times H\left(I_{x,y_{\mathrm{mCherry}}}-I_{B}\right)\;$$

using Equation (5).

The area of intended gene expression, *A_I_*, was determined by multiplying the total number of activated pixels in cascade blue dextran (CBD) fluorescence images with the area of a single pixel, *A*_pix_ (1.68 × 10^−6^ mm^2^)

(7) $$A_{I}=\sum_{x=1}^{x_{\max}}\sum_{y=1}^{y_{\max}}H\left(I_{x,y_{\mathrm{CBD}}}-I_{\mathrm{CBD}}\right)\times A_{\mathrm{pix}}\;$$

where $I_{x,y_{\mathrm{CBD}}}$ are the pixel values of cascade blue dextran fluorescence images and *I*_CBD_ = 500 a.u. was set as an appropriate threshold to capture the outline of the area comprising of droplets containing cascade blue dextran and, hence, arabinose and αHL. The same step function was used (see Equation (5)).

The area of unintended gene expression, *A*_U_, was calculated by multiplying the total number of pixels outside of *A*_I_ with *A*_pix_

(8) $$A_{U}=\sum_{x=1}^{x_{\max}}\sum_{y=1}^{y_{\max}}\left[H\left(I_{x,y_{\mathrm{mCherry}}}-I_{B}\right)\;\times \;H\left(I_{\mathrm{CBD}}-I_{x,y_{\mathrm{CBD}}}\right)\right]\times A_{\mathrm{pix}}\;\;$$

The area of no gene expression, *A*_N_, was calculated by multiplying the total number of pixels within *A*_I_ with *A*_pix_

(9) $$A_{N}=\sum_{x=1}^{x_{\max}}\sum_{y=1}^{y_{\max}}\left[H\left(I_{x,y_{\mathrm{mCherry}}}-I_{B}\right)\;\times \;H\left(I_{x,y_{\mathrm{CBD}}}-I_{\mathrm{CBD}}\right)\right]\times A_{\mathrm{pix}}\;\;$$

*PF* was computed by using Equation (1).

For bacterial interference competition, PF was determined as described above, with sfGFP as the read‐out for induced gene expression, as opposed to mCherry. Here, a threshold value of ≥200 a.u. was used, which was determined from the baseline expression of sfGFP as a consequence of colicin E7 baseline expression levels in bacteria without the presence of arabinose.

### Volume‐to‐Surface‐Area Ratio (*R*_VSA_)

First, the area of intended gene expression, *A*_I_, of a droplet network was determined by multiplying the number of cascade blue dextran pixels reaching the threshold of 500 a.u. with *A*_pix_. Based on *A*_I_, the pixel size (mm^2^) and the number of droplets per layer, the cross‐sectional area of a single droplet was determined (mm^2^). This information was used to determine the volume of a single droplet (mm^3^) and the volume of a droplet network based on the total number of droplets (mm^3^). The volume‐to‐surface‐area ratio (*R*_VSA_) was then determined as shown in Equation (2), yielding a value (mm) describing the relation between volume and area of intended gene expression, *A*_I_.

### Magnetic Handles for Guided Landing of Droplet Networks

Magnetic handles were prepared by adding a preheated (60 °C) solution (composition not stated by manufacturer) containing Ni‐particles (MagneHis Ni‐Particles, Promega) to preheated (60 °C) M9 medium containing 1.5% w/v of ULGA at a volume ratio of 2:3. Droplets (50–250 µm diameter) were then ejected (FemtoJet 4x, Eppendorf) into lipid‐in‐oil (same composition used for printing of droplet networks) and cooled at 4 °C for 35 min. The gelled droplets were transferred next to the corners of a droplet network by pipetting and pushed onto the droplet network to which they adhered through DIB formation. The droplet network was then placed on top of a bacterium‐laden hydrogel, under which a magnet had been placed to direct the orientation of the droplet network.

### Heat‐Induced Dehydration of Droplet Networks

Droplet networks were printed in SOG cuvettes, which contained lipid‐in‐oil solution (500 µL). The cuvettes were then placed in the center of a transparent heating plate (Leica Thermo Plate), which was set to 36 or 43 °C. The temperature in the lipid‐in‐oil solution was allowed to reach equilibrium as determined with a sensor (Thorlabs, TSP01). The printing solution was diluted with Milli‐Q water so that the concentrations of the components in the droplet networks would be as desired after shrinkage (36 or 43 °C). Once the desired decrease in volume was attained as determined by microscopy, a shrunken network was brought to room temperature for 30 min before transfer on top of the bacterium‐laden hydrogel.

### Droplet Networks as Switchable Modules

Two kinds of droplet networks were printed separately; one acted as a storage module and the other as a release module. Functional droplets in storage modules (containing 33 × 10^−3^ м arabinose, 50 µg mL^−1^ αHL and 250 × 10^−6^ м cascade blue dextran; cyan compartments, Figure 5) were connected with functional droplets in release modules (containing 50 µg mL^−1^ αHL and 250 × 10^−6^ м cascade blue dextran; yellow compartments, Figure 5). Storage and release modules were connected by gently pushing them together in lipid‐in‐oil solution by using a pipet. Once the droplet networks were connected correctly, such that arabinose could diffuse from the storage modules through αHL pores to release modules, gene expression was induced in bacterial cells according to the pattern formed by the functional droplets of the release module at the *xy*‐plane (e.g., a stripe‐like pattern, Figure 5d).

### Definitions and Calculations

Mean Gene Expression *I*_M_: Mean gene expression (mCherry or sfGFP) refers to the sum of all pixel values in activated pixels divided by the total number of activated pixels, where pixels are considered activated when baseline expression levels are reached (see Pattern Fidelity (PF) Quantification by Image Analysis).

Total Gene Expression *I*_T_: Total gene expression (mCherry or sfGFP) refers to the sum of all pixel values in activated pixels, where pixels are considered activated when baseline expression levels are reached (see Pattern Fidelity (PF) Quantification by Image Analysis).

Starting Ratio: Starting ratio refers to the ratio between cells of one over the other genotype in a bacterial community at the start of an experiment when the two genotypes were mixed homogeneously in an ULGA hydrogel solution (*t* = 0 h).

Bacterial competition: Bacterial competition refers to the process by which one individual decreases the survival or reproduction of others.^[^ ^86^ ^]^

### Statistical Analysis

Epi‐fluorescent microscopy images were processed according to “Pattern Fidelity Quantification by Image Analysis”, which was visualized in Figure 2a. The data is presented as mean ± standard deviation (SD) and the number of replicates is stated in each figure caption. For analysis of statistical differences between groups it was first tested whether the data was normally distributed using the Shapiro–Wilk test (*p* < 0.05). Given groups were normally distributed, significance between groups was tested performing a two‐sample *t*‐test. If data of at least one group was not normally distributed, significance between groups was tested by performing a Wilcoxon rank‐sum test. *p < 0.05, **p < 0.01, ***p < 0.001 and ****p < 0.0001.

## Conflict of Interest

The authors declare no conflict of interest.

## Supporting information



Supporting Information

## Data Availability

The data that support the findings of this study are available in the Supporting Information of this article.
