# Supplementary material for: High‐Resolution Patterned Delivery of Chemical Signals From 3D‐Printed Picoliter Droplet Networks
Source: Adv Mater. 2025 Apr 30;37(28):2412292. doi: 10.1002/adma.202412292 (PMC12272041; doi:10.1002/adma.202412292)
Supplement: Supplementary file 1 — Supporting Information [file ADMA-37-2412292-s001.docx]

Supporting Information

**High-resolution Patterned Delivery of Chemical Signals from 3D-printed Picoliter Droplet Networks**

*Jorin Riexinger*, Thomas Caganek, Xingzao Wang, Yutong Yin, Khoa Chung, Linna Zhou, Hagan Bayley* and Ravinash Krishna Kumar**

**Table of Contents**

**Supplementary Notes 1**

Supplementary Note 1: Arabinose-induced Gene Expression: pBAD Rationale 1

Supplementary Note 2: Pattern Fidelity as a Measure of Gene Expression Controllability 1

Supplementary Note 3: Magnetic Beads for Guiding Tissue Placement 2

Supplementary Note 4: *E. coli* Interference Competition 4

Supplementary Note 4.1: Colicin Biology 4

Supplementary Note 4.2: Differences Between Competing Strains 4

Supplementary Note 4.3: Testing Growth Inhibition using Agar Overlay Assay 5

Supplementary Note 4.4: Actively Dividing Cells Are Required for Colicin Competition 6

Supplementary Note 4.5: Droplet Networks and Colicin Competition 7

Supplementary Note 4.6: Importance of Culture Medium for Colicin Competition 8

Supplementary Note 4.7: Local Lysis 9

Supplementary Note 4.8: Colicin E2 Promoter as Indication of DNA Damage 10

**Supplementary Figures 13**

Figure S1: Plasmid maps 13

Figure S2: Magnetic beads guide landing of droplet networks 14

Figure S3: Comparison between droplet networks with and without reservoirs 16

Figure S4: Overlay assay of E7-inducible against colicin E7, colicin E8 and susceptible cells 17

Figure S5: Co-culture of E7-inducible and susceptible cells. 18

Figure S6: Time-dependent release of arabinose from droplet networks 19

Figure S7: Importance of culture medium for colicin competition 21

Figure S8: Localized expression of colicin E7 underneath droplet networks 22

Figure S9: E7-inducible causes DNA damage in susceptible cells 24

Figure S10: Localized DNA damage in susceptible cells by arabinose release from droplet networks 26

**Supplementary Tables 27**

Table S1: Summary of strains and plasmids 27

**Supplementary Methods 28**

Growth Overlay Assay 28

Propidium Iodide Staining 28

Determination of Colony Forming Units (CFU) 28

**References 30**

**Supplementary Notes**

**Supplementary Note 1: Arabinose-induced Gene Expression: pBAD Rationale**

We chose pBAD as an inducer system for multiple reasons: 1) The permeability of arabinose (chemical signal) was minimal (Figure 1(h)) through lipid bilayers (comprising of a lipid composition 2:1 (molar ratio) DPhPC:POPC) formed between compartments within droplet networks (droplet interface bilayers = DIBs) and between droplet networks and the hydrogel (droplet hydrogel bilayers = DHBs). This allowed us to control the arabinose flux from the droplet networks into the bacterium-laden hydrogels by changes in both arabinose and αHL concentration, and prevented diffusion of arabinose between compartments within the droplet networks unless αHL was present in the DIBs. 2) pBAD systems have been reported to behave as an all-or-nothing system,^[1]^ which we reasoned would be ideal to achieve patterned gene expression based on arabinose flux from droplet networks. We hypothesized that below or above a critical arabinose concentration, the gene expression in cells would be low and high, respectively, rather than a gradient going from low to high. Hence, a tight population gene expression pattern could be achieved depending on the arabinose gradient released over time. This all-or-nothing system works once a critical concentration of arabinose is reached, resulting in high expression levels of genes downstream of the P_BAD_ promoter, such as *mCherry* or *cxE7*. At the same time, increased activity of the transcriptional regulator AraC induces the expression of *araE*, which encodes for the arabinose transporter AraE. This increases the uptake of arabinose, leading to a positive feedback loop that rapidly increases protein expression in the cells until a maximum is reached.

**Supplementary Note 2: Pattern Fidelity as a Measure of Gene Expression Controllability**

As described, our *PF* measure describes the controllability of gene expression in *E. coli* populations by comparing the area of unintended gene expression, *A_U_*, to the area of intended gene expression, *A_I_*. Another measure we considered was factoring *A_N_*, which refers to the area within *A_I_*, where no gene expression was induced. However, this measure was not chosen to optimize arabinose release from 3D-printed droplet networks, as we did not encounter areas within *A_I_* where gene expression was not induced (unless droplets did not form DHBs with the hydrogel (see Figure 3(g))). Therefore, our *PF* measure was chosen to quantify gene expression in areas outside of the intended gene expression area as a measure over controlled release of arabinose. However, in addition we reported values of *A_N_* (Figure 2(g)), which takes into account areas within *A_I_* where insufficient DHB formation as a consequence of printing defects and irregularities of the surface of bacterium-laden hydrogels prevented arabinose release.

By using cascade blue dextran to reveal where arabinose was released from droplet networks, we could determine the area of unintended gene expression, *A_U_* and *A_N_* (Figure 2(a)). However, in some cases, the definitive location of arabinose release was imperfect due to imaging limitations.^[2]^ This is because the layers of droplets in our droplet networks are stacked by shifting every other layer in both the *x*- and *y*-directions such that hexagonally-packed structures can form. So, droplets of the same position in *x* and *y* within the droplet networks were offset depending on the layer. Therefore, accurate determination of the droplets from which arabinose was being released into the bacterium-laden hydrogel was difficult in some cases. In particular, this may cause inaccuracies in locating fine patterns, such as single-droplet diffusive pathways (Figure 4(b)) or patterns in mask layers, where the fluorescence signal from cascade blue dextran in droplets comprising reservoir layers may interfere with the fluorescence signal from cascade blue dextran droplets in the mask layers (Figure S4 and S5).

**Supplementary Note 3: Magnetic Beads for Guiding Tissue Placement**

Our 3D droplet printing technology can be time-consuming when printing intricate patterns. This is because, 1) droplets are positioned one after another, 2) when printing droplets of different compositions (e.g. with or without arabinose and αHL), two printing nozzles are used of which the printing stage has to be moved between the nozzle positions. For example, printing droplet networks composed of 10 × 10 × 8 droplets (in *xyz*-direction) takes 67 minutes for a cross-like pattern and 87 minutes for an arrow-like pattern. To overcome this, we reasoned that only the bottom layers (masks) in the droplet networks are necessary to be patterned, as long as this ‘mask’ layer was connected to a reservoir of arabinose and αHL-containing droplets to supply arabinose to the mask layers. Therefore, we printed cubic, arabinose-containing ‘reservoirs’ (4-8 layers) on top of the mask layers (4 layers) connecting reservoirs to the mask via αHL-mediated droplet diffusive pathways. Using this method, we reduced our printing time significantly, for example, the printing time for droplet networks composed of twelve cross-like patterned layers (9 × 9 × 12 droplets) was 56 minutes, while the printing time for droplet networks composed of 4 mask layers (9 × 9 × 4 droplets) of the same pattern and 8 reservoir layers (7 × 7 × 8) on top was 41 minutes. Moreover, this method stored arabinose more efficiently within the droplet network in terms of occupied volume, as 8 reservoir layers were composed of a total of 392 αHL and cascade blue dextran-containing droplets as opposed to 136 in 8 patterned layers.

To avoid reservoir droplets from accidentally rolling to the patterned bottom layer of droplet networks during printing and, hence, disrupting intended patterns, reservoirs were designed to be slightly smaller than bottom layers (e.g. 6 × 6 droplets (reservoir) instead of 8 × 8 droplets (mask)). As a result, we found that droplet networks predominantly flipped before they landed on top of the bacterium-laden hydrogel (i.e. the reservoir interfaced with the hydrogel instead of the mask layer, Figure S2(a)). Out of 45 droplet networks composed of patterned masks and a reservoir, 88.9% flipped during the transfer and landed with the reservoir facing the hydrogel. In this case, arabinose would diffuse directly from the reservoir to the cells, resulting in gene expression patterns reflecting the shape of the reservoir, rather than the intended pattern (Figure S2(b)). This occurred likely due to hydrodynamic forces in the viscous lipid-in-oil solution, aligning the smaller reservoir towards the hydrogel. To overcome the flipping, we developed a mechanism to control the landing of droplet networks on top of the hydrogel. This comprised of attaching droplets containing magnetic beads (1.5% w/v ultra-low gelling agarose and nickel magnetic beads) to the corners of the mask layers before the transfer and then placing a magnet underneath the bacterium-laden hydrogel to guide the mask layer towards the hydrogel. Using this method, the correct landing was achieved in 76.5% of 34 transferred droplet networks, allowing for patterned gene expression in bacterial cells by the mask layers rather than the reservoir layers (Figure S2(c)).

Next, we investigated the number of mask layers that were necessary to consistently induce patterned gene expression. We printed droplet networks composed of 1, 2, 3 and 4-layered masks comprising a stripe-like pattern and an 8-layered reservoir on top (33 mм arabinose and 50 μg mL^-1^ αHL). Accordingly, 4 connected mask layers were necessary to induce stripe-like gene expression in bacterial cells (Figure 3(d)-Figure 3(g) and Figure S2(d)).

Finally, we compared gene expression patterns induced by arabinose release from droplet networks with and without reservoirs. Droplet networks with reservoirs consisted of 4-layered masks encoding for a single-droplet pathway and 4-layered reservoirs on top (Figure S3(a)), whereas droplet networks without reservoirs consisted of 8-layered masks encoding for a single-droplet pathway. Using both types of droplet networks (with and without reservoirs), single-droplet gene expression patterns could be induced. Further, we found that there was no significant difference between droplet network type regarding *PF* and mean mCherry expression (Figure S3(b)), confirming that droplet networks composed of masks and reservoirs are not only more efficient in terms of printing time and occupied space but also induced gene expression at similar spatial resolution compared to droplet networks comprised completely of mask layers.

**Supplementary Note 4: *E. coli* Interference Competition**

Supplementary Note 4.1: Colicin biology

*E. coli* can produce colicins, protein toxins that target susceptible *E. coli* strains, which is crucial for interference competition between strains.^[3,4]^ These colicins, specifically group A colicins (E7, E8), are encoded on a plasmid (pCol, type I plasmids: 6–10 kb) with about 20 copies per cell. Type I plasmids carry group A colicins, which parasitize the Ton system in *E. coli* for entry into the periplasm. Group A colicins include nuclease colicins (E7, E8) causing DNA damage to susceptible cells which mostly cause cell death.^[5]^

On the pCol plasmid, the colicin operon is controlled by the LexA protein, which represses the SOS promoter. The SOS response, triggered by DNA damage, upregulates RecA, enabling LexA self-cleavage. This allows RNA polymerase binding to the SOS promoter for colicin operon transcription, leading to colicin expression. Nuclease colicins are associated with two genes: one encoding the colicin and the other the cognate immunity protein. The immunity protein is constitutively expressed, preventing self-intoxication. Last, the operon also contains a gene that encodes the lysis protein needed for colicin release.^[5]^

Supplementary Note 4.2: Differences Between Competing Strains

We utilized six distinct strains in our inducible competition assays:

1. S (BZB1011): a susceptible strain to DNA damage (and as a consequence, likely death) by colicins E7 and E8. It does not carry a colicin-producing plasmid (Table S1, 2)
2. S-GFP (BZB1011 pUA66-PcolE2::gfp): a susceptible strain to DNA damage (and as consequence, likely death) by colicins E7 and E8. It also does not carry a colicin-producing plasmid but, but harbours a DNA damage-reporting plasmid (Table S1, 5)
3. E7-inducible (BZB1011 pKC1-*PBAD*:-*ColE7*-AMP): a strain that upregulates the colicin E7 operon (toxin, immunity, and lysis proteins) in the presence of arabinose, and is susceptible to colicin E8 (Table S1, 3)
4. E7R-inducible (BZB1011 pYY1-*PBAD*:-*ColE7*-*mCherry*-AMP): a strain that upregulates the colicin E7 operon (toxin, immunity, and lysis proteins) and the mCherry protein in the presence of arabinose, and is susceptible to colicin E8 (Table S1, 4)
5. E7 (BZB1011 pColE7): the natural colicin E7-producing strain that upregulates the colicin E7 operon when experiencing DNA damage, and is susceptible to colicin E8 (Table S1 , 6)
6. E8 (BZB1011 pColE8): the natural colicin E8-producing strain that upregulates the colicin E8 operon when experiencing DNA damage, and is susceptible to colicin E7 (Table S1, 7).

Notably, colicin-producing strains (E7-inducible, E7R-inducible, E7 and E8) possess additional mechanisms for toxin release. These strains exhibit a basal toxin production rate. For E8, approximately 1 in 200 cells stochastically upregulate toxin and lysis protein production^[5]^; the basal rate is assumed to be lower for the E7-inducible strain. Specifically for the natural colicin E8 producer, E8 can amplify toxin production through autoinduction. This density-dependent mechanism involves clonemates being more likely to upregulate their colicin E8 operon when in the proximity of an E8 cell that releases toxins. This occurs because E8 produces lower amounts of cognate immunity protein so that the probability of DNA damage is increased upon import of a clonemates’ released colicin E8.^[5]^

Supplementary Note 4.3: Testing Growth Inhibition Using Agar Overlay Assay

To test whether our E7-inducible (BZB1011 pKC1-*PBAD*:-*ColE7*-AMP) strain expressed colicin E7 upon induction with arabinose, we performed overlay assays (Figure S4(a)-(d), see Supplementary Methods), where E7-inducible cells were spotted on top of cells that natively produce colicin E7 (BZB1011 pColE7 – E7), (Table S1, 6) or colicin E8 (BZB1011 pColE8 – E8), (Table S1, 7) and susceptible cells (BZB1011 – S), (Table S1, 2). Using an agar overlay assay^[6]^ when E7-inducible was spotted on top of an E7 top agar, E7-inducible continued to grow when no arabinose was added to the plates. Adding arabinose concentrations of 0.5% (w/v), 1% (w/v) and 5% (w/v) to the agar caused complete lysis of E7-inducible. In contrast, E7 continued to grow at all arabinose concentrations used (0% (w/v) to 5% (w/v)), due to it expressing the E7 immunity protein (Figure S4(a)).

When E7-inducible was spotted on E8 top agar, no growth was observed of E7-inducbile after 18 h (Figure S4(b)). We reasoned this was because of the higher basal expression of E8 compared to E7-inducible, and E8’s ability to respond to an attack from E7 as the natural operon is upregulated by DNA damage. Taken together E8 is dominant because it can produce a lot more toxin than the uninduced E7-inducible strain (see colicin biology section).

With arabinose concentrations of ≥0.5% (w/v) in the agar, E7-inducible was activated, leading to not only mass lysis of E7-inducible (as the entire colicin operon, including the lysis protein is under control of the P_BAD_ promoter), but also growth inhibition in native colicin E8-expressing cells, because of the high concentration of colicin E7 released from E7-inducible cells (Figure S4(b)).

In the case where E7-inducible was spotted on top of LB agar containing susceptible cells (BZB1011 – S), E7-inducible continued to grow when no arabinose was present. However, a halo around the location where E7-inducible was spotted, presumably because of low basal levels of E7 expression in LB medium, affecting susceptible cells at the boundary of the E7-inducible spot (Figure S4(c)). In contrast, when E7-inducible was spotted on top of M9 agar containing susceptible cells (BZB1011 – S), both E7-inducible and S cells grew next to each other when no arabinose was present (Figure S4(d)). As soon as arabinose was added to the plates (≥ 0.5%(w/v)) E7-inducible was activated, leading to growth inhibition of susceptible cells both in LB agar plates (Figure S4(c)) and in M9 medium agar plates (Figure S4(d)).

Supplementary Note 4.4: Actively-Dividing Cells Are Required for Colicin Competition

Next, we investigated whether growth of susceptible cells can be inhibited by induced expression of colicin E7 at a range of arabinose concentrations (0 mм to 333 mм) when both cells were mixed homogeneously at an equal starting ratio (1:1) in M9 ultra-low gelling agarose (ULGA, 1.5% w/v). At a total starting cell density of 3.6 × 10^9^ cells mL^-1^ the mean GFP expression of E7-inducible, arising from constitutive expression of GFP, decreased only slightly with increasing arabinose concentrations (Figure S5(a)), indicating a low number of lysis events and, hence, minimal expression of colicin E7. This is supported by the fact that the mean RFP expression of susceptible cells, arising from constitutive expression of RFP, did not decrease significantly with increasing arabinose concentrations (Figure S5(a)). We hypothesized that colicin E7 expression was low because not many cell divisions of the E7-inducible strain occurred in the hydrogel at these high cell densities.

To confirm whether lower cell densities, and hence whether sustained cell divisions during interference competition is required for E7-inducible to express colicin E7 in M9 ULGA, we decreased the starting cell density of E7-indicuble to 1.6 × 10^7^ cells mL^-1^ within the hydrogel. Indeed, the number of micro-colonies arising from E7-inducible decreases with increasing arabinose concentrations within the M9 ULGA gels, suggesting induced expression of colicin E7 lysis protein at lower cell densities (Figure S5(b)). Similarly, when both E7-inducible and susceptible cells were mixed homogeneously at equal starting ratio and a combined starting cell density of 1.6 × 10^7^ cells mL^-1^ the number of E7-inducible micro-colonies decreased with increasing arabinose concentrations. No micro-colonies were observed of susceptible cells after 18 hours of co-culture, which suggests that baseline expression levels of colicin E7 in the absence of arabinose was sufficient to inhibit the growth of susceptible cells (Figure S5(c)). Hence, the homogeneous distribution of E7-inducible and susceptible cells in our bacterium-laden hydrogel enhanced the effect of colicin E7 baseline expression compared to the agar overlaying assay.

Therefore, we decreased the starting ratio from initially 1:1 to 1:9 and 1:99 (number of E7-inducible cells to number of susceptible cells). At a starting ratio of 1:9 and total starting cell density of 1.6 × 10^7^ cells mL^-1^ both E7-inducible and susceptible cells grew to homogeneously-distributed micro-colonies within M9 ULGA gels when no arabinose was present (Figure S5(d)). With increasing arabinose concentrations both the number of E7-inducible and susceptible micro-colonies decreased, suggesting both lysis of E7-inducible and DNA-damaging effects on S cells. At a starting ratio of 1:99 of E7-inducible to susceptible cells, less susceptible cells were inhibited presumably because of the much lower starting densities of E7-inducible and hence lower release concentrations colicin E7 (Figure S5(e)).

Supplementary Note 4.5: Droplet Networks and Colicin Competition

From our above results, we hypothesized that arabinose released from droplet networks into bacterium-laden M9 ULGA gels (supplemented with 24 mм glucose) could induce localized lysis of E7-inducible cells that would, in turn, clear a localized area of susceptible cells when the two strains were incubated at a starting ratio of 1:9. Therefore, we placed droplet networks containing 33 mм arabinose and 50 μg mL^-1^ αHL on top of bacterium-laden hydrogels containing E7-inducible and S cells. Droplet networks were placed either immediately after the gels were formed or 24 h after the gels were formed. However, when droplet networks were place after the gels were formed, the number of micro-colonies of both E7-inducible and susceptible cells did not change significantly, indicating that arabinose release from droplet networks did not induce levels of colicin E7 required for clearing of susceptible cells. The time point of tissue placement in regards to formation of the gel (just after or 24 hours after formation) did not affect the final number of E7-inducible and S cells (Figure S6(a) and (b)).

We reasoned higher concentrations of arabinose within droplet networks were required to induce sufficient expression of colicin E7 in E7-inducible. However, a ten-fold increase in arabinose concentration (333 mм) did not lead to lysis of E7-inducible within 42 h of the competition assay against S cells, as confirmed by the number of micro-colonies arising from E7-inducible cells (Figure S6(c)). Therefore, the number of micro-colonies arising from susceptible cells did not change significantly when comparing droplet networks with or without arabinose (Figure S6(d)).

Supplementary Note 4.6: Importance of Culture Medium for Colicin Competition

We wondered whether the culture medium in the gels had a significant impact on induced gene expression of E7-inducible. In particular, we hypothesized that glucose, which was supplemented to M9 as a carbon source throughout previous experiments, could act as catabolic repressor, limiting gene expression levels.^[7,8]^ Our hypothesis was based on the differences in basal expression of E7-inducible from overlay assays (Figure S4(c) and S4(d)), where without addition of arabinose E7-inducible visibly inhibited and did not visibly inhibit S cells when LB and M9 medium were used, respectively. To test these effects of media composition on colicin expression in E7-inducible, we monitored cell lysis in E7-inducible populations in ULGA gels (at a starting cell density of 3.6 × 10^9^ cells mL^-1^) composed of M9 supplemented with 24 mм glucose, M9 supplemented with 24 mм glycerol or LB at arabinose concentrations of 0 mм, 6 mм, 33 mм and 66 mм. We monitored cell lysis by tracking constitutive sfGFP expression (on the chromosome of E7-inducible), as and indication of cell viability, and propidium iodide staining in E7-inducible, as indication of cell death, which has previously been shown to confirm cell lysis.^[3]^ A decrease in sfGFP expression and increase in propidium iodide fluorescence would indicate cell lysis (as propidium iodide can only penetrate cells and bind to DNA when the membrane is compromised). While the mean gene expression of sfGFP did not change significantly when comparing glucose and glycerol as supplemented carbon source in M9 medium, a significant decrease in sfGFP was observed for gels composed of LB with increasing arabinose concentrations (Figure S7(a)). Moreover, propidium iodide fluorescence increased when cells were in LB medium supplemented with arabinose as compared to M9 medium, indicating increased cell lysis (Figure S7(b)). The combination of decreased sfGFP expression and increased propidium iodide intensity indicated a significant reduction in number of micro-colonies formed with increasing arabinose concentrations. Interestingly, the propidum ioide fluorescence was significantly increased for gels composed of M9 supplemented with glycerol at arabinose concentrations of 33 mм and 66 mм compared to gels supplemented with glucose, indicating increased cell lysis in the presence of glycerol as compared to glucose (Figure S7(b)).

Supplementary Note 4.7: Local Lysis

Next, we investigated whether E7-inducible cells could be lysed when droplet networks containing 333 mм of arabinose and 50 μg mL^-1^ of αHL monomer were placed on top of bacterium-laden hydrogels composed of LB ULGA. For this we formed gels containing E7-inducible at a starting cell density of 1.6 × 10^7^ cells mL^-1^ and measured the number of micro-colonies in the periphery and center when droplet networks were or were not placed on top of the bacterium-laden hydrogels (Figure S8(a) and Figure S8(b)). Center refers to the area directly underneath droplet networks and the same areas when no droplet networks were placed, whereas periphery refers to the area outside of where droplet networks were placed (Figure S8(a)). So, droplet networks were used as reference region of interest both when droplet networks were placed on top of the bacterium-laden hydrogel (+ST) or when droplet networks were not placed on top(−ST). Critically, the cell density of E7-inducible cells was significantly reduced both in the periphery and center when droplet networks were placed on top of the bacterium-laden hydrogel compared to when no droplet networks were present (Figure S8(b)), suggesting that local lysis of E7-inducible is indeed possible when arabinose is released from droplet networks in LB.

As the localized expression of colicin E7 was achieved in LB, we investigated the effect of toxin release on susceptible cells at different starting ratios. Here, the total starting cell density was kept constant at 1.6 × 10^7^ cells mL^-1^, while altering the starting ratio of E7-inducible to susceptible cells from 1:9 to 9:1. We found that the relative abundance of E7-inducible decreased when droplet networks containing 333 mм of arabinose and 50 μg mL^-1^ of αHL monomer were placed on top of the bacterium-laden hydrogel (Figure S8(c)). However, the cell density of susceptible cells was not affected significantly when droplet networks were placed on top of the bacterium-laden hydrogel as compared to when they were not. Taken together, these results indicated that the initial assumption of distinctive cell clearing of susceptible cells as a consequence of induced E7 expression may be limited within the time course of our experiments.

Supplementary Note 4.8: Colicin E2 Promoter as Indication of DNA Damage

From our experiments, we thought a susceptible strain was needed to report on DNA damage as an indication of cell inhibition, rather than focusing on cell clearing. We reasoned that the natural promoter of colicin-expressing strains, which is regulated by DNA damage (see colicin biology section), could act as reporter of DNA damage in susceptible cells. Therefore, we transformed susceptible cells with a reporter plasmid (pUA66-P*colE2*:*sfgfp*)^[3,4]^ which allowed us to monitor the activity of P_colE2_ based on GFP expression levels and, hence, levels of DNA-damage that susceptible cells may experience as a consequence of induced colicin E7 expression. In summary, susceptible cells (S-GFP) would express GFP in response to DNA-damage. Additionally, to track colicin operon activity in the E7-inducible strain, we created a reporter strain with *mCherry* downstream of the colicin E7 lysis gene, i.e. when the operon was active, fluorescent protein would be produced (BZB1011 pYY1-*PBAD*:-*ColE7*-*mCherry*-AMP, E7R-inducible).

First, we mixed E7R-inducible cells and S-GFP cells at equal starting ratio and a total starting cell density of 1.6 × 10^7^ cells mL^-1^, or S-GFP cells only at a starting cell density of 0.8 × 10^7^ cells mL^-1^, in M9 ULGA gels supplemented with 24 mм of glycerol and a range of arabinose concentrations (0 mм to 8 mм, Figure S9(a)). After 18 hours of culture at 37°C we found that mCherry expression increased with increasing arabinose concentrations, indicating increasing levels of colicin E7 expression with increasing concentrations of arabinose (Figure S9(b)). The mean GFP expression in S-GFP cells increased significantly at arabinose concentrations of 0.25 mм and 0.5 mм in the gels as compared to when no arabinose was present in the gels. However, a further increase of arabinose (>0.5 mм) concentration caused a significant drop in GFP expression (Figure S9(b)), of which we assumed cells were killed too quickly (with high concentrations of expressed colicin E7) to report on DNA-damage. To correlate GFP expression with the ability of S-GFP cells to replicate, we re-suspended cells from the ULGA gels and plated them on selective LB agar plates (with kanamycin selecting for S-GFP cells) and measured the relative number of colony forming units (*R_CFU_*) of S-GFP cells after 12 hours at 37°C:

$R_{CFU}=\frac{N_{CFU}}{N_{{CFU}_{single}}}100\%$, (S1)

where $N_{CFU}$ refers to the number of colony forming units at a certain concentration of arabinose in the gels before plating (0 mм to 8 mм) and $N_{{CFU}_{single}}$ refers to the number of colony forming units when only S-GFP cells were seeded in ULGA gels without E7R-inducible cells and without arabinose (single). We found a significant drop in *R_CFU_* at an arabinose concentration of 0.5 mм in the ULGA gels, confirming that the observed increase in GFP indeed was an indication of cell inhibition of S-GFP cells.

From this, we printed droplet networks containing 50 μg mL^-1^ of αHL monomer and a range of arabinose concentrations (0 mм to 33 mм) and placed them on top of homogeneously-distributed cells of E7R-inducible and S-GFP cells at equal starting ratio and a total cell density of 1.6 × 10^7^ cells mL^-1^. We did not observe any DNA damage in S-GFP cells when droplet networks contained ≤3 mм of arabinose. However, at 16 mм of arabinose the number of micro-colonies of S-GFP cells that experienced DNA damage increased significantly, both underneath the droplet network and in the periphery. Further increase in arabinose (33 mм) led to a decrease in the number of micro-colonies expressing sfGFP, suggesting decreased viability, i.e. cells were inhibited too quickly to report on DNA-damage (Figure S9(c)).

In order to achieve patterned DNA damage in S-GFP cells, we adjusted both arabinose (8 mм, 12 mм and 16 mм) and αHL monomer concentrations (0 μg mL^-1^, 10 μg mL^-1^, 25 μg mL^-1^ and 50 μg mL^-1^) so to control the release of arabinose from droplet networks. With increasing arabinose and αHL concentrations the number of S-GFP cells experiencing DNA-damage increased both underneath droplet networks (Figure S10(a)), and outside of where droplet networks were placed (Figure S10(b)). Moreover, the mean sfGFP expression increased with increasing arabinose and αHL concentrations (Figure S10(c)), while the mean micro-colony 2D cross-sectional area of S-GFP cells increased up to 198.5 µm^2^ before it decreased to 63.7 µm^2^ (Figure S10(d)).

**Supplementary Figures**

**Figure S1: Plasmid maps**


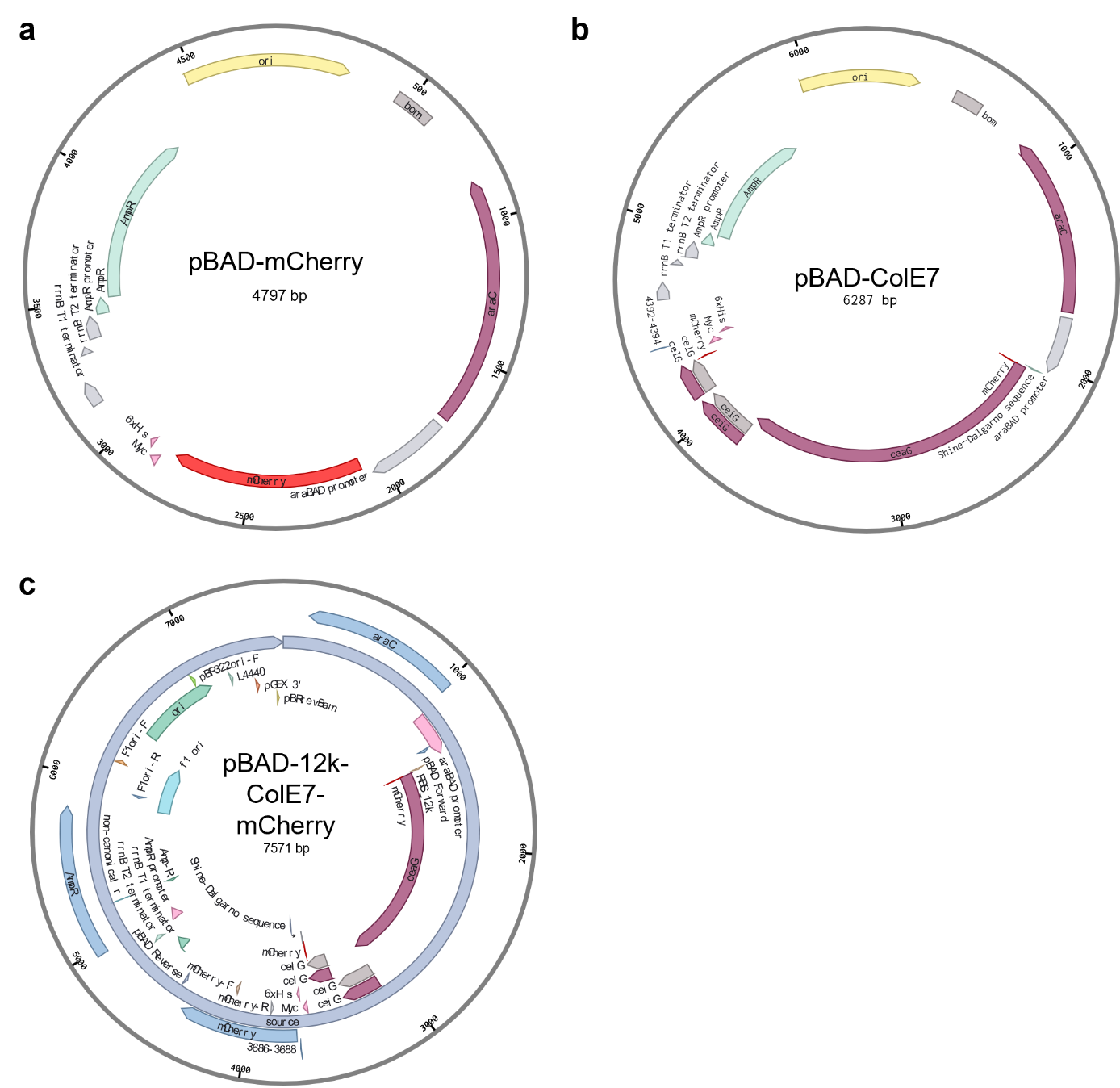


**Figure S1.** Plasmid maps of plasmids used throughout this work. (a)-(c) Schematics of plasmid maps of pJS1-*PBAD*:-*mCherry*-AMP, pKC1-*PBAD*:-*ColE7*-AMP and pYY1-*PBAD*:-*ColE7*-*mCherry*-AMP, respectively. Plasmid maps were created using Benchling.^[9]^

**Figure S2: Magnetic beads guide landing of droplet networks**


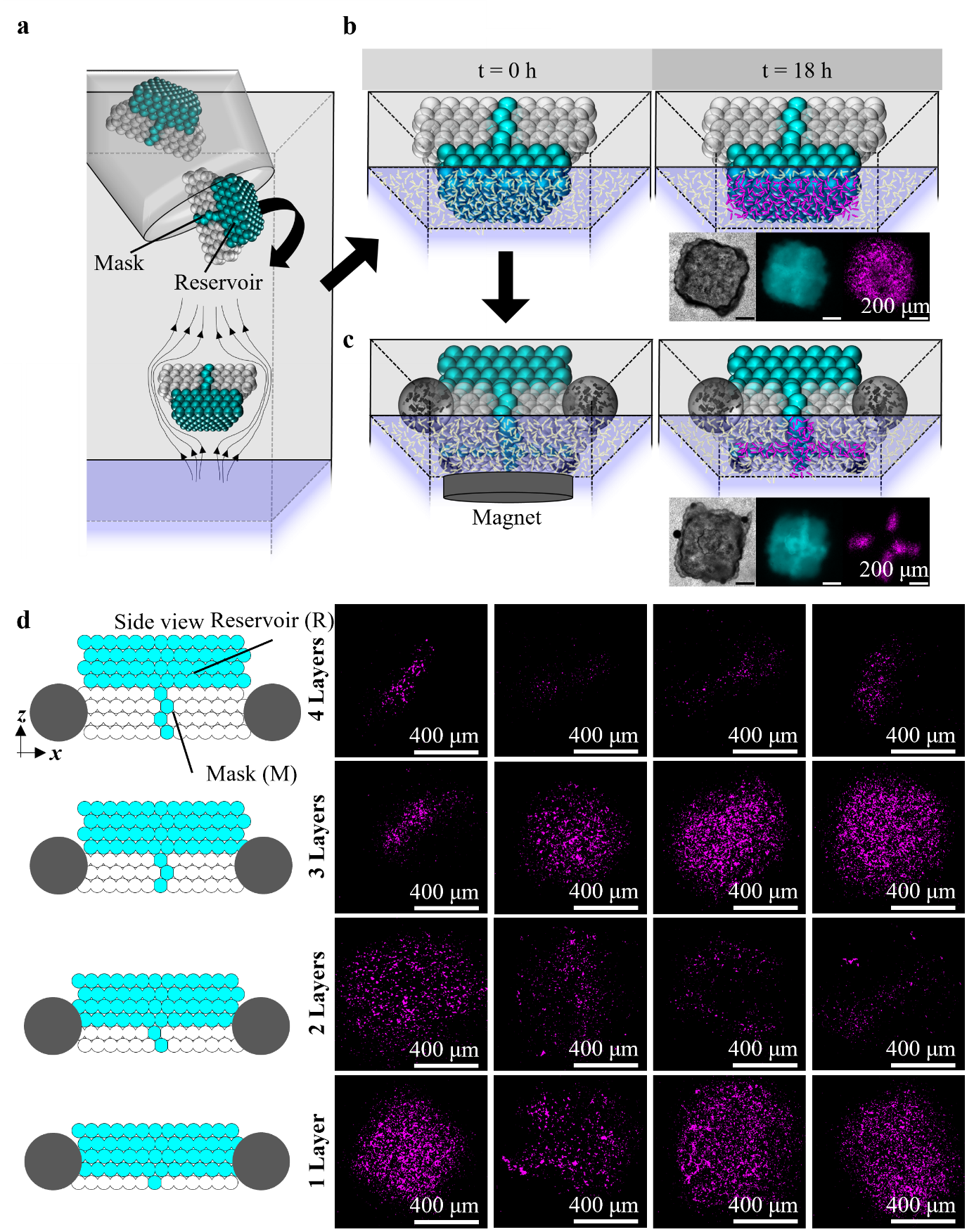


**Figure S2.** Patterned gene expression using masks and reservoirs incorporated into 3D-printed droplet networks. (a) Schematic of a droplet network (that contains a mask and a reservoir) flipping during placement on top of a bacterium-laden hydrogel, causing the droplet network to predominantly land with the reservoir directly contacting the bacterium-laden hydrogel. Arrows around the sinking droplet network represent oil displacement, which we reasoned drives the flipping process as the reservoir is smaller in size compared to the mask. (b) Schematic of a droplet network that flips and lands reservoir side down activating expression of mCherry in the bacterial population. Bottom right microscopy images (bright-field and epi-fluorescence) is an example of a droplet network landing reservoir side down onto the bacterium-laden hydrogel. Images were taken 18 hours after placement of the droplet network (containing 33 mм arabinose and 50 μg mL^-1^ αHL monomer). Cyan fluorescence represents cascade blue dextran, while magenta fluorescence represents mCherry expression. (c) Schematic of a droplet network that does not flip and lands mask side down due to magnetic beads (droplets composed of 1.5% w/v ultra-low gelling agarose and nickel magnetic beads) attached to each corner of the mask of a droplet network and a magnet placed underneath the bacterium-laden hydrogel during tissue transfer. The release of arabinose from the mask of a droplet network containing 33 mм arabinose and 50 μg mL^-1^ αHL monomer activated patterned (cross-like shape) expression of mCherry in the bacterial population. Bottom right microscopy images (bright-field and epi-fluorescence) is an example of a droplet network landing mask side down onto the bacterium-laden hydrogel. Images were taken 18 hours after droplet network placement and cyan and magenta fluorescence represent cascade blue dextran and mCherry expression, respectively. (a)-(c) In droplet networks cyan droplets contained 33 mм arabinose, 50 μg mL^-1^ αHL monomer, and 250 μм cascade blue dextran, whereas grey droplets did not contain these components. (d) Epi-fluorescent microscopy images depicting mCherry expression (magenta) induced by the release of arabinose from droplet networks composed of 1, 2, 3 or 4 mask layers (stripe-like pattern) and 4 reservoir layers 18 hours after tissue placement on top of the bacterium-laden hydrogel. The droplets within the stripe-like pattern of the mask and all reservoir droplets contained 33 mм arabinose, 50 μg mL^-1^ αHL monomer, and 250 μм cascade blue dextran, whereas droplets of mask layers located outside the stripe-like pattern did not contain these components. Shown are n = 4 replicates.

**Figure S3: Comparison between droplet networks with and without reservoirs**


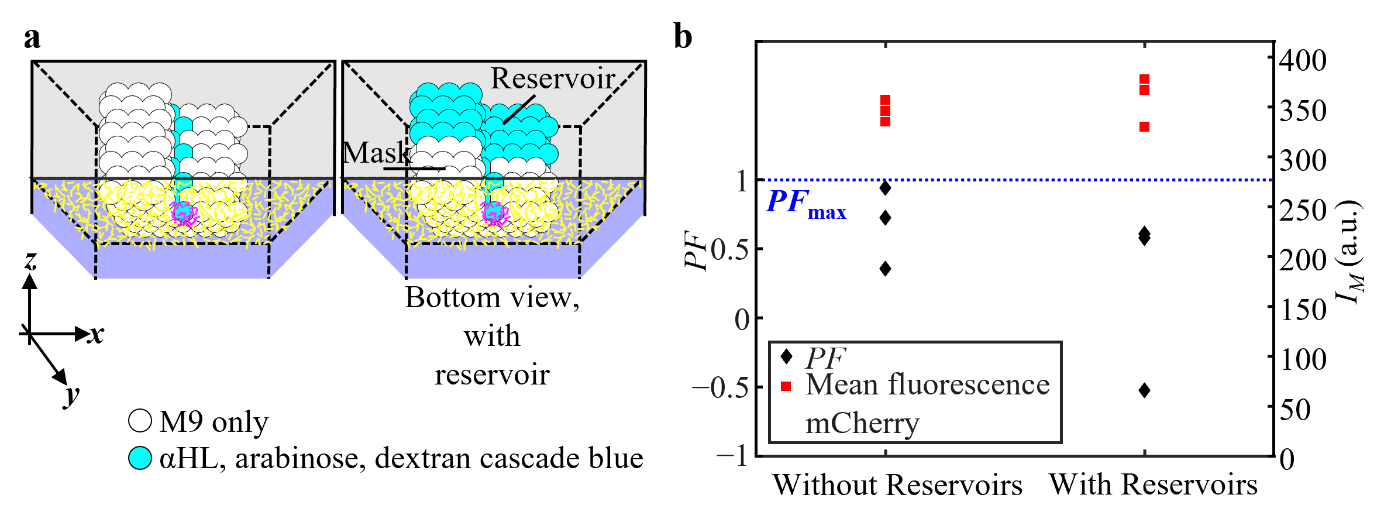


**Figure S3.** Single-droplet gene expression with and without reservoirs. (a) Schematic illustrating droplet networks composed of 8 patterned layers (without reservoirs) or 4 patterned mask layers and 4 reservoir layers (with reservoirs). Both types of droplet networks were composed of droplets that contained 33 mм arabinose and 50 μg mL^-1^ αHL monomer (cyan droplets) or did not contain these components (white droplets). (b) Graph of the *PF* and mean mCherry expression induced by arabinose release from droplet networks with and without reservoirs on top of masks comprising of a single-droplet diffusive pathway that contacts the hydrogel surface forming a DHB.

**Figure S4: Overlay assay of E7-inducible against colicin E7, colicin E8 and susceptible cells**


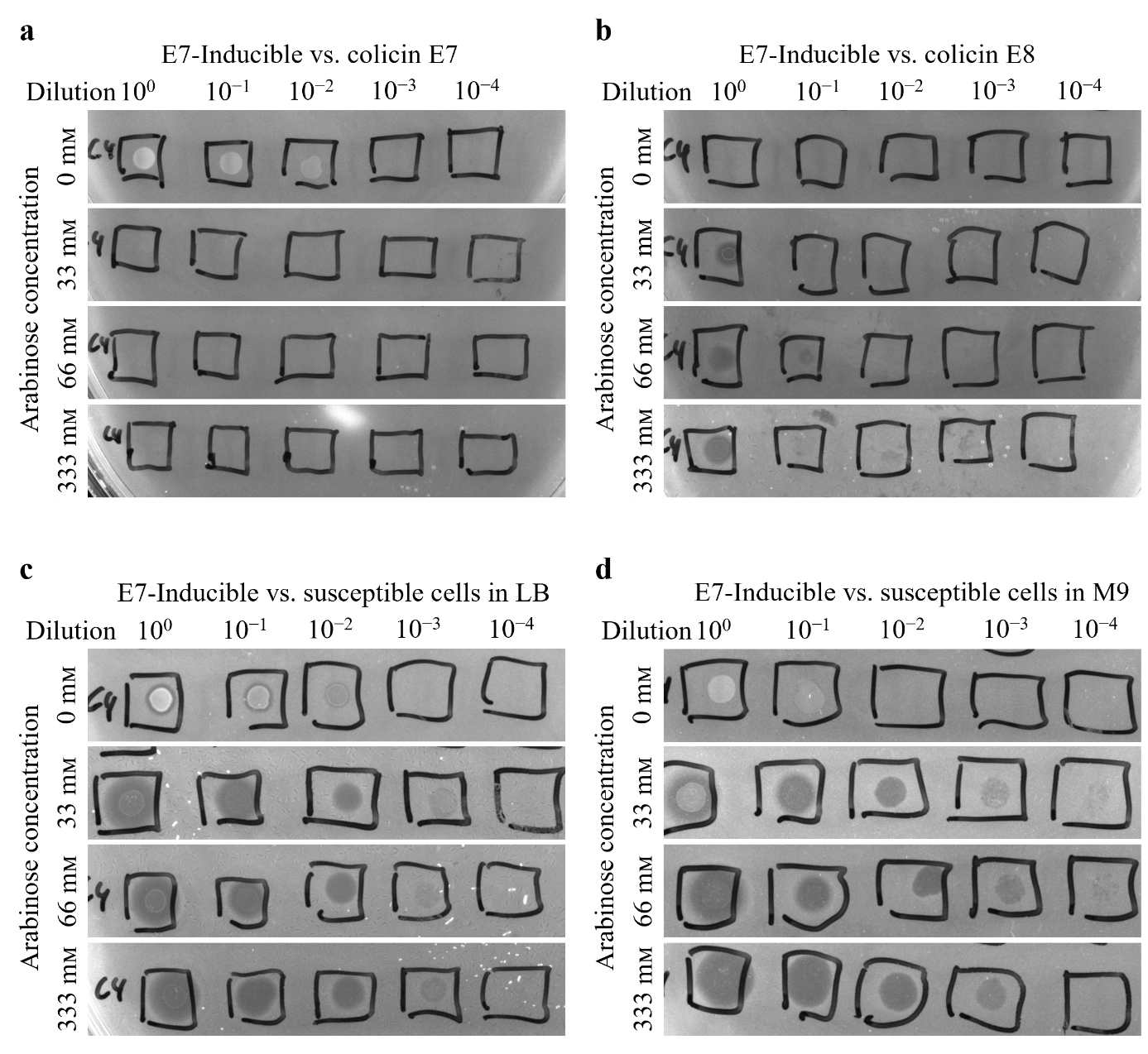


**Figure S4.** Overlay assay of E7-inducible against colicin E7, colicin E8-expressing and susceptible cells. (a) – (d) Photographs of serial dilutions from 10^0^ to 10^−4^ (of an overnight liquid culture) of E7-inducible (BZB1011 pBAD-E7) spotted on top of agar plates containing E7 cells (BZB1011 pColE7) (a), E8 cells (BZB1011 pColE8) (b), or susceptible cells (BZB1011) (c) and (d), after 12 hours at 37°C where the agar contained between 0 mм and 333 mм arabinose. The agar plates were composed of 1.5% (w/v) of agar in LB (a)–(c) or M9 (d).

**Figure S5: Co-culture of E7-inducible and susceptible cells.**


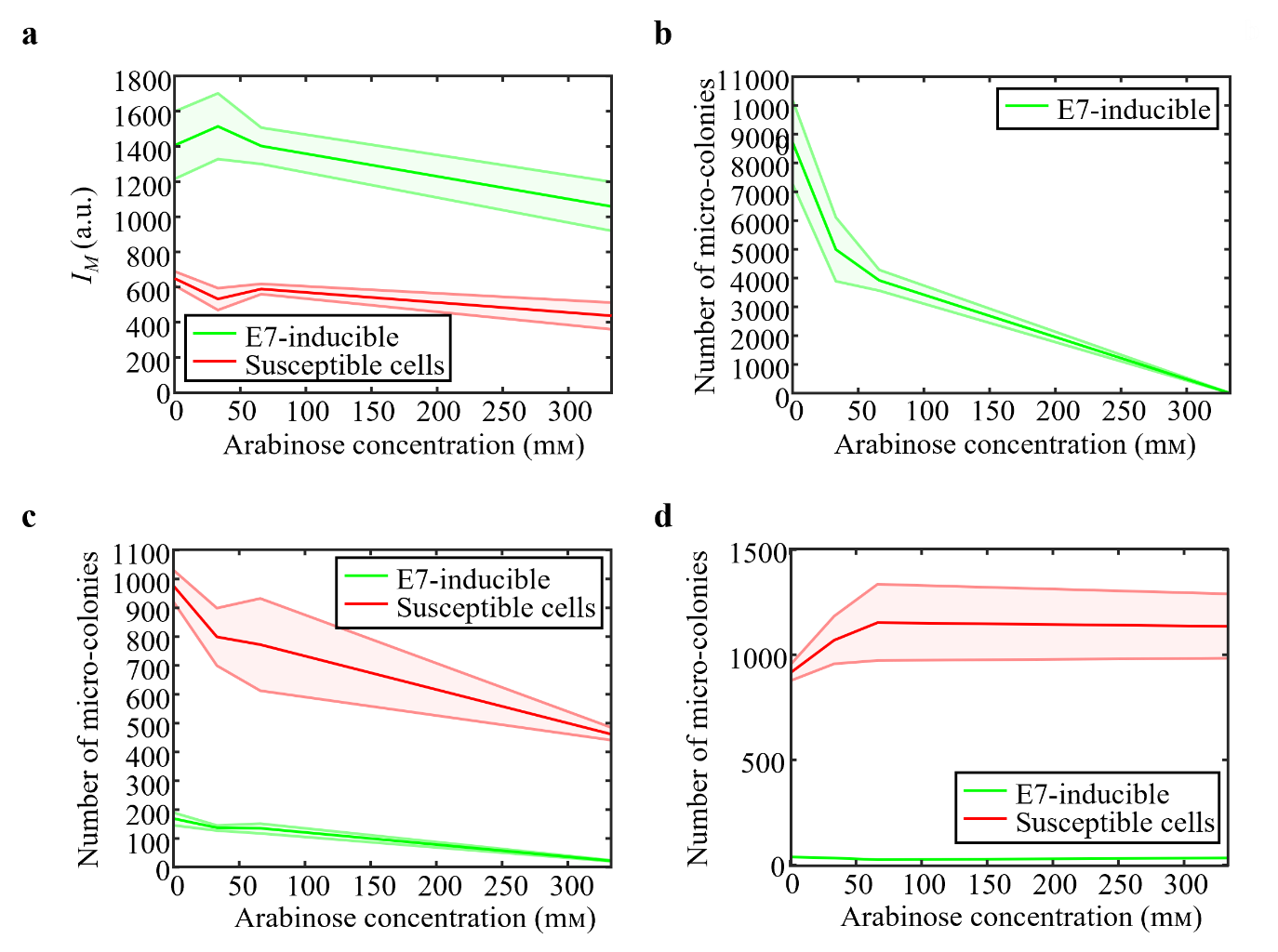


**Figure S5.** Co-culture of E7-inducible and susceptible cells. (a) Graph of the mean GFP and RFP expression of E7-inducible (green line) and S cells (red line), respectively, mixed homogeneously at equal starting ratios in M9 ultra low gelling temperature agarose (ULGA) gels at a combined cell starting density of 3.6 × 10^9^ cells mL^-1^ at varying arabinose concentrations after 18 hours at 37°C. (b) Graph of the number of micro-colonies formed of E7-inducible in M9 ULGA gels after 18 h at 37°C at varying arabinose concentrations at a cell starting ratio of 1.6 × 10^7^ cells mL^-1^. (c)-(e) Graphs of the number of micro-colonies formed of E7-inducible (green line) and S cells (red line) mixed homogeneously at starting ratios of 1:1 (equal number between E7-inducible and S cells), 1:9 or 1:99 (E7-inducible to S cells), respectively, in M9 ultra low gelling temperature agarose (ULGA) gels at a combined cell starting density of 1.6 × 10^7^ cells mL^-1^ at varying arabinose concentrations after 18 hours at 37°C. The solid line and shaded area represent the mean and standard deviation of n = 4 technical replicates.

**Figure S6: Time-dependent release of arabinose from droplet networks**


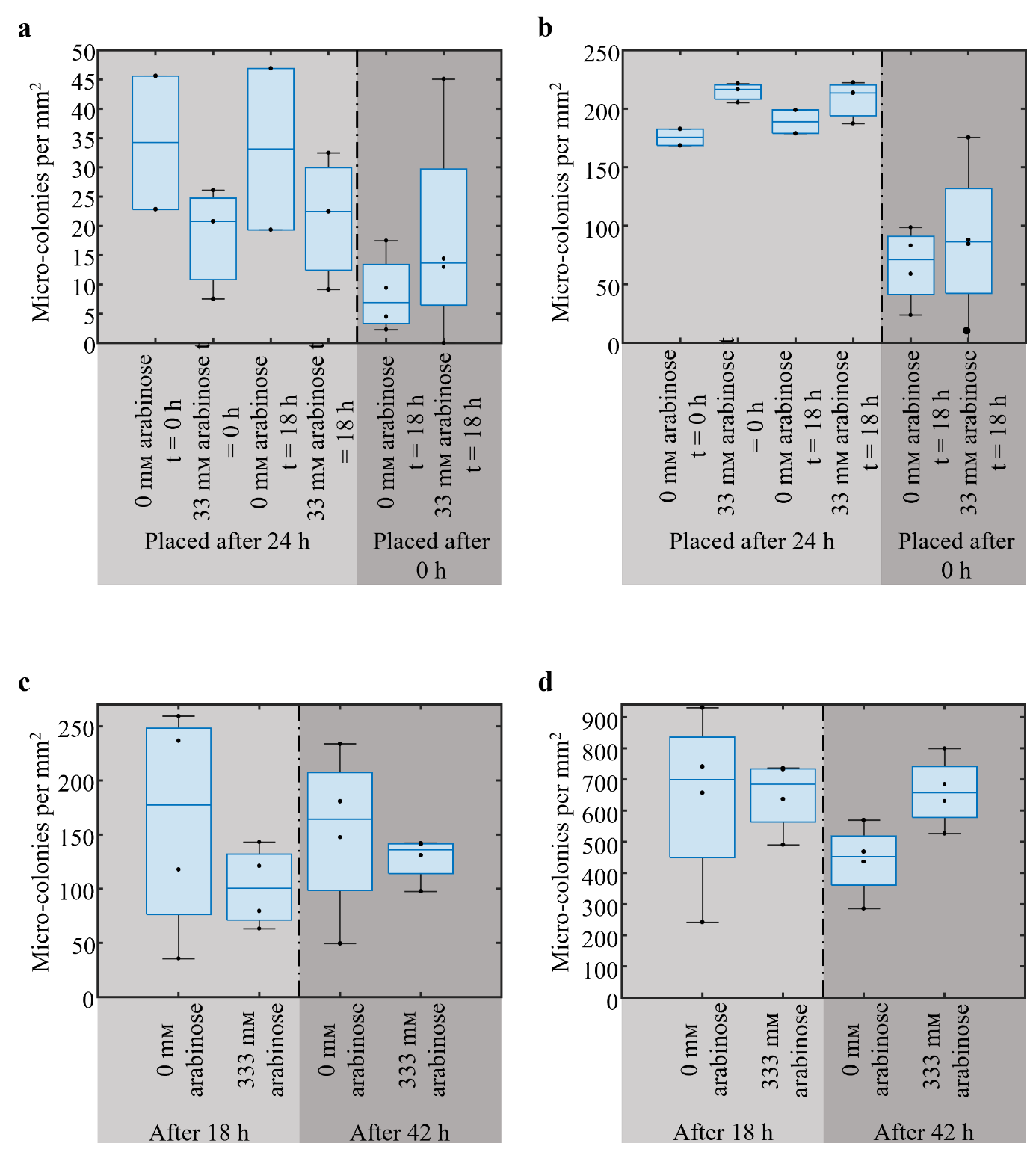


**Figure S6.** Time-dependent release of arabinose from droplet networks. (a) and (b) Box plots of the number of E7-inducible (BZB1011 pBAD-E7) (a) and susceptible cells (BZB1011) (b) arising from competition between them, when droplet networks containing either 0 mм or 33 mм arabinose and 50 μg mL^-1^ αHL were placed on top of the bacterium-laden hydrogel for 18 hours after placement immediately after gel formation (placed after 0 h) or placement after 24 hours of cell growth (placed after 24 h). The starting ratio was 1:9 (E7-inducible to susceptible cells) while the total starting cell density was 1.6 × 10^7^ cells mL^-1^. Individual data points on the box plots are technical replicates. (c) and (d) Box plots of the number of E7-inducible (BZB1011 pBAD-E7) (c) and susceptible cells (BZB1011) (d) arising from competition between them, when droplet networks containing 333 mм arabinose and 50 μg mL^-1^ αHL were placed on top of the bacterium-laden hydrogel for 18 or 42 hours, respectively. The starting ratio was 1:9 (E7-inducible to susceptible cells) while the total starting cell density was 1.6 × 10^7^ cells mL^-1^. Individual data points on the box plots are technical replicates.

**Figure S7: Importance of culture medium for colicin competition
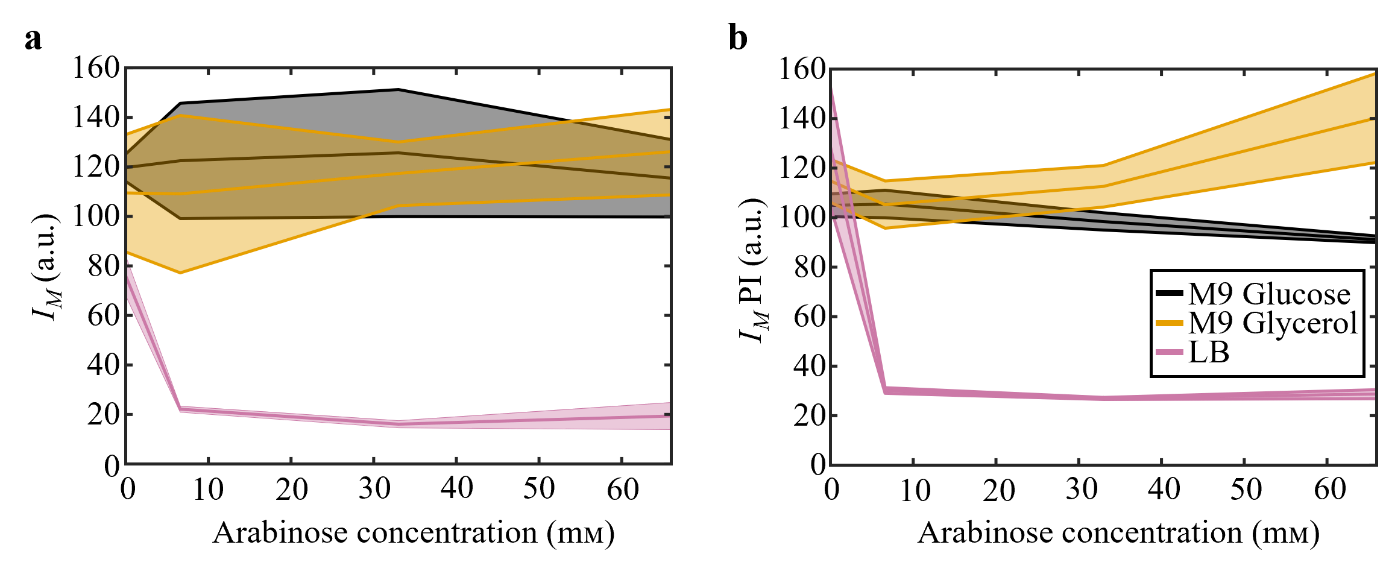
**

**Figure S7.** Importance of culture medium for colicin competition. (a) and (b) Graphs of the mean sfGFP expression (a) and mean intensity of intracellular propidium iodide (b), in ULGA gels composed of M9 supplemented with 24 mм glucose, M9 supplemented with 24 mм glycerol or LB at a range of arabinose concentrations (0 mм, 6 mм, 33 mм and 66 mм) after 18 hours at 37°C. The gels contained E7-inducible cells at a starting cell density of 3.6×10^9^. The solid line and shaded area represent the mean and standard deviation of n = 4 technical replicates.

**Figure S8: Localized expression of colicin E7 underneath droplet networks**


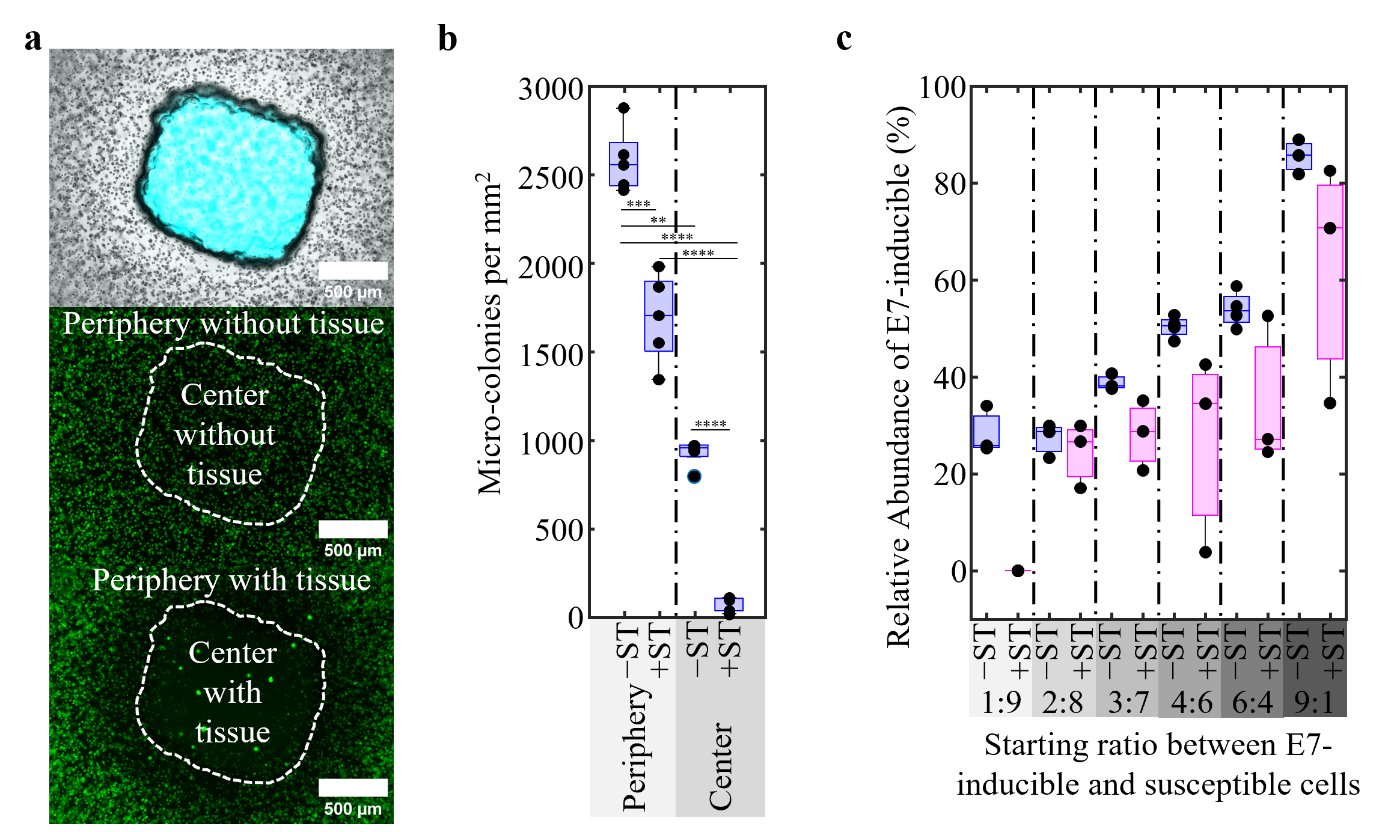


**Figure S8.** Localized expression of colicin E7 underneath droplet networks. (a) Composite microscope image (bright-field and cascade dextran blue, top image) of a droplet network on top of a bacterium-laden hydrogel and sfGFP fluorescence images of bacterium-laden hydrogel when a droplet network was placed (bottom image) and was not placed on top of the image (center image). The white dashed line represents the outline of the tissue, which was used as a reference area for when no droplet network was placed on top of the bacterium-laden hydrogel. The bacterium-laden hydrogels contained E7-inducible cells (BZB1011 pBAD-E7) at a starting cell density of 1.6 × 10^7^ cells mL^-1^. (b) Boxplot of the micro-colonies per mm^2^ within the peripheral and central areas of E7-inducible cells (BZB1011 pBAD-E7) (**Figure S1**(c) and **Table S1**) seeded at a starting cell density of 1.6 × 10^7^ cells mL^-1^ in ULGA gels composed of LB medium at 37°C for 18 hours after droplet networks containing 333 mм arabinose and 50 μg mL^−1^ αHL monomer were (+ST) and were not (−ST) placed on top of the bacterium-laden hydrogels. The individual data points are depicted for each condition with n = 5 technical replicates. It was tested whether the data was normally distributed using the Shapiro-Wilk test (*p* < 0.05). Given groups were normally distributed, significance between groups was tested performing a two-sample t-test. If data of at least one group was not normally distributed, significance between groups was tested by performing a Wilcoxon rank-sum test. ***p* < 0.01, ****p* < 0.001 and *****p* < 0.0001. (c) Boxplot of the relative abundance (RA) of E7-inducible micro-colonies at a range of starting ratios (1:9 to 9:1, ratio between E7-inducible cells and susceptible cells) when droplet networks containing 333 mм arabinose and 50 μg mL^-1^ of αHL monomer were placed (+ST) or not placed (-ST) on top of the bacterium-laden hydrogel after 18 hours at 37°C. The total starting cell density was 1.6 × 10^7^ cells mL^-1^. Shown are individual data points of n = 4 technical replicates and boxplots are mean and interquartile range.

**Figure S9: E7-inducible causes DNA damage in susceptible cells**


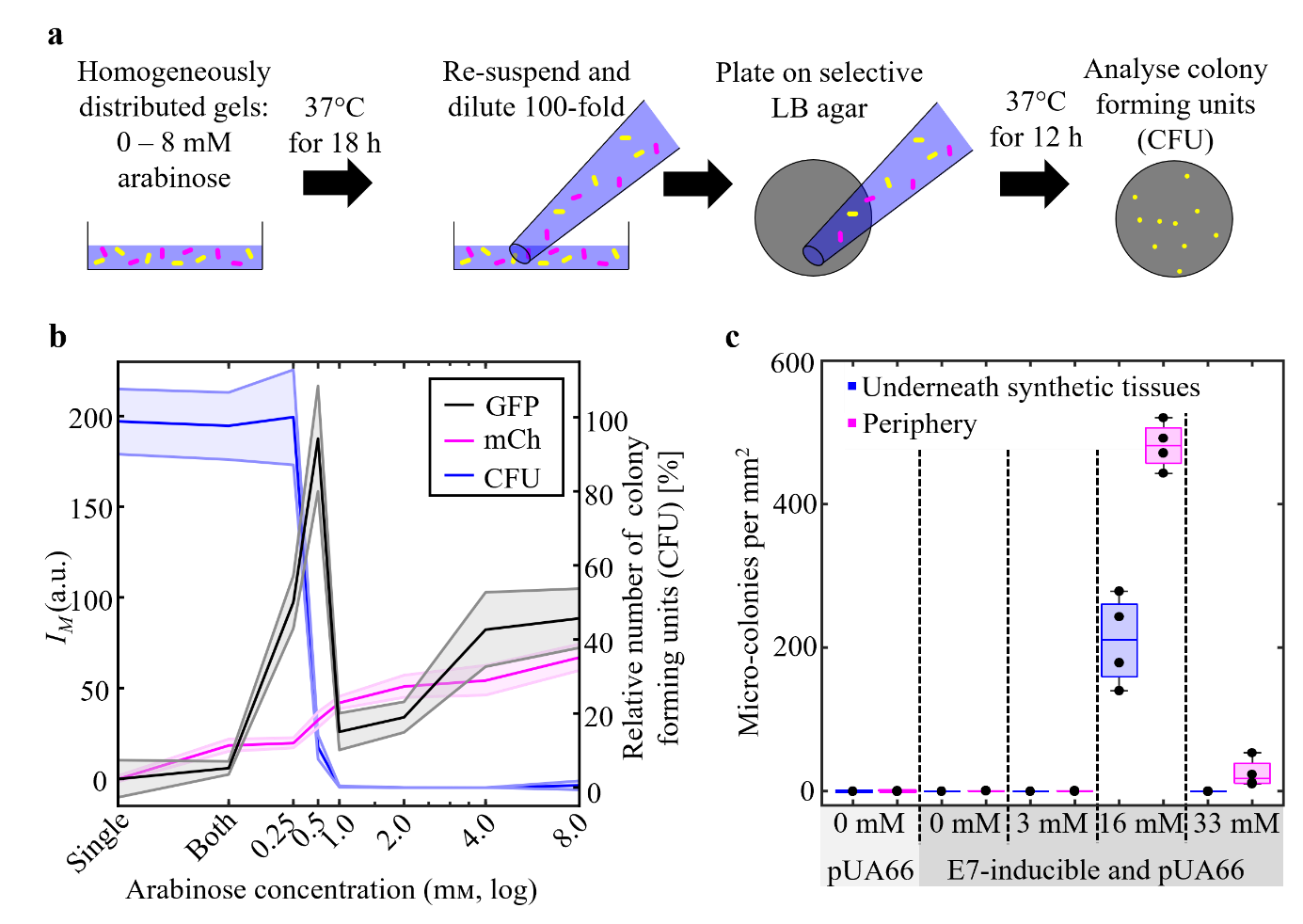


**Figure S9.** E7-inducible causes DNA damage in susceptible cells. (a) Schematic depicting the workflow to characterize the correlation between GFP expression and the relative colony forming units (CFU), whereby E7R-inducible or E7R-inducible and S-GFP cells are grown for 18 h at 37 °C in M9 ULGA gels, before re-suspension and plating on LB agar plates containing 50 μg mL^-1^ of kanamycin to select for colony-forming units arising from S-GFP cells. (b) Graph of the mean mCherry expression (mean fluorescence intensity of activated pixels, indicating colicin E7 expression), mean sfGFP expression (indicating DNA damage in susceptible cells), and the relative number of colony forming units (CFU) from susceptible cells over a range of arabinose concentrations (0 mм to 8 mм) added to bacterium-laden hydrogels composed of M9 ULGA supplemented with 24 mм glycerol. The starting ratio between E7R-inducible and susceptible cells (BZB1011, S-GFP cells) was 1:1 at a total starting cell density of 1.6 × 10^7^ cells mL^-1^. Mean mCherry expression and mean GFP expression of E7-inducible and susceptible cells, respectively, were measured after 18 hours of co-culture, while the number of colony forming units from susceptible cells was measured 12 hours after solubilization of the M9 ULGA gels and plating on selective medium. ‘Single’ indicates M9 ULGA gels containing only susceptible cells seeded at 0.8 × 10^7^ cells mL^-1^ without the addition of arabinose (0 mм), while ‘both’ indicates the co-culture of E7-inducible and susceptible cells at an equal starting ratio and a total starting cell density of 1.6 × 10^7^ cells mL^-1^ without the addition of arabinose (0 mм). The solid line and shaded area represent the mean and standard deviation of n = 4 technical replicates. (c) Boxplots of the number of micro-colonies per mm^2^ of activated susceptible cells (expressing GFP as a consequence of DNA-damage) in areas underneath the droplet networks containing a range of arabinose concentrations (0 mм – 33 mм) and 50 μg mL^-1^ of αHL monomer, and peripheral areas (outside of where the droplet networks were placed) at 18 hours after placement of droplet networks at 37°C. E7R-inducible (BZB1011, E7R-inducible) and susceptible cells (BZB1011, S-GFP cells) were seeded at a 1:1 starting ratio and a total starting cell density of 1.6 × 10^7^ cells mL^-1^. The ULGA gels contained M9 medium supplemented with 24 mм glycerol. Shown are individual data points of n = 4 technical replicates and boxplots display the mean and interquartile range.

**Figure S10: Localized DNA damage in susceptible cells by arabinose release from droplet networks**


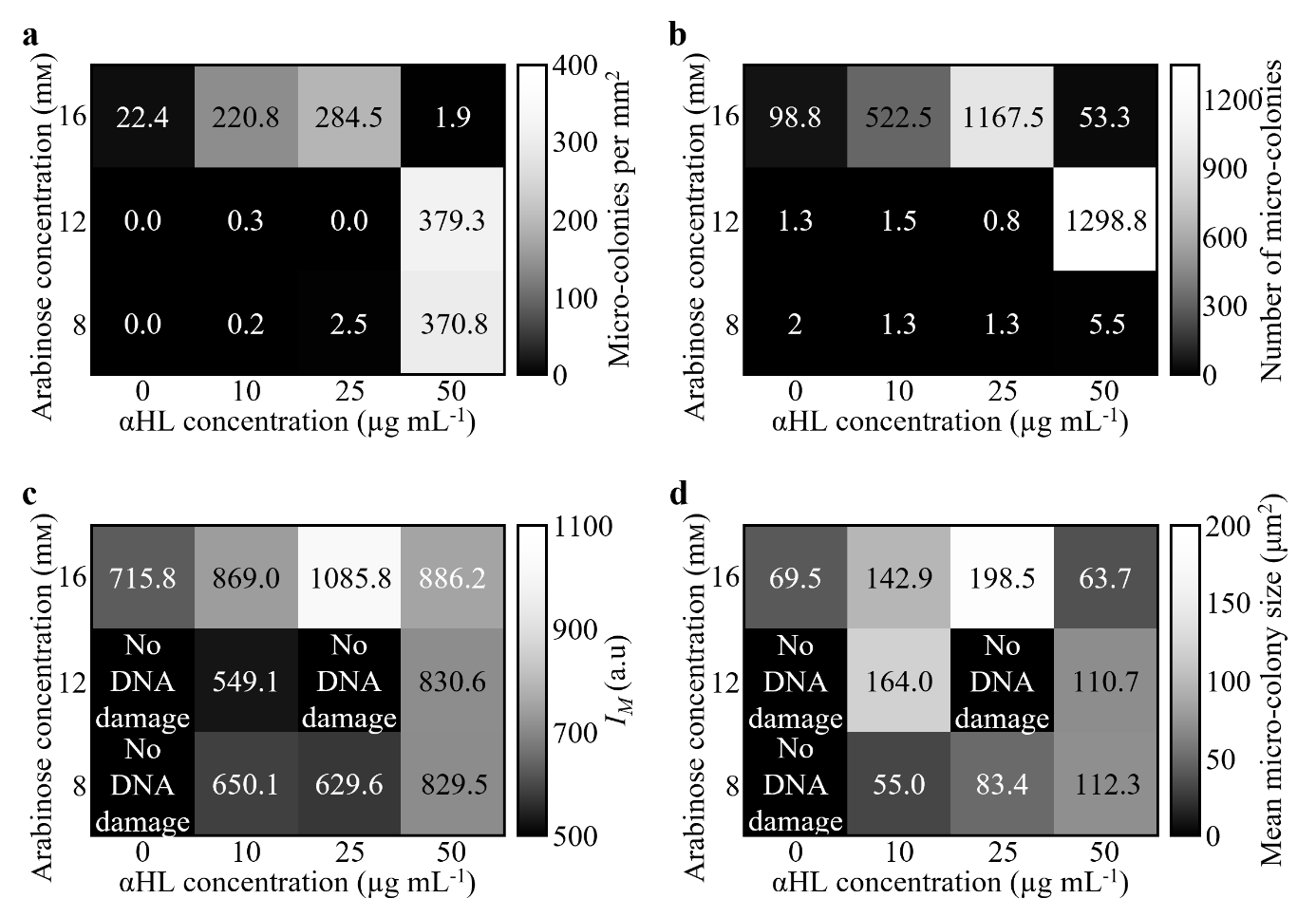


**Figure S10.** Localized DNA damage in susceptible cells. (a)-(d) Heat-maps of micro-colonies per mm^2^ in areas underneath droplet networks (a), the total number of micro-colonies in peripheral areas outside of where droplet networks were placed (b), the mean GFP expression (c) and mean micro-colony size (d) of activated susceptible cells (S-GFP cells experiencing DNA-damage caused by colicin E7 expressed from E7R-inducible) after 18 hours at 37°C. E7R-inducible cells (BZB1011, E7R-inducible) and susceptible cells (BZB1011, S-GFP) were seeded at equal starting ratio and a total cell density of 1.6 × 10^7^ cells mL^-1^. The ULGA gels were composed of M9 supplemented with 24 mм glycerol. Droplet networks contained a range of arabinose concentrations (8 mм, 12 mм and 16 mм) and a range of αHL monomer concentrations (0 μg mL^-1^, 10 μg mL^-1^, 25 μg mL^-1^ and 50 μg mL^-1^).

**Supplementary Tables**

**Table S1: Summary of strains and plasmids**

**Table S1.** Summary of strains and plasmids.

|  | **Strain and genotype** | **Recombinant DNA** | **Abbreviation** | **Promoter** | **Description** | **Source of recombinant DNA** |
| --- | --- | --- | --- | --- | --- | --- |
| **1** | BZB1011  *Pmax:sfgfp::Tn7* | pJS1-*PBAD*:-*mCherry*-AMP | mCherry-inducible | pBAD | Constitutive expression of sfGFP, induced expression of mCherry | This study |
| **2** | BZB1011 *Pmax:mrfp1::Tn7* | - | S | - | Constitutive expression of RFP | - |
| **3** | BZB1011 *Pmax:sfgfp::Tn7* | pKC1-*PBAD*:-*ColE7*-AMP | E7-inducible | pBAD | Constitutive expression of sfGFP, induced expression of colicin E7 | This study |
| **4** | BZB1011 *Pmax:sfgfp::Tn7* | pYY1-*PBAD*:-*ColE7*-*mCherry*-AMP | E7R-inducible | pBAD | Constitutive expression of sfGFP, induced expression of colicin E7 and mCherry | This study |
| **5** | BZB1011 | pUA66-*PcolE2::sfgfp* | S-GFP | pColE2 | Unlabeled, GFP expression upon sensed DNA-damage | ^[3,4]^ |
| **6** | BZB1011 *Pmax:mrfp1::Tn7* | pColE7 | pcolE7 | SOS | Constitutive expression of RFP, natural colicin E7 plasmid | ^[10]^ |
| **7** | BZB1011 *Pmax:mrfp1::Tn7* | pColE8 | pcolE8 | SOS | Constitutive expression of RFP, natural colicin E8 plasmid | ^[10]^ |

**Supplementary Methods**

**Growth overlay assay**

Plates for growth inhibition assay consisted of two layers of solidified agar. The bottom layer did not contain cells, while the top layer contained bacterial cells. LB/M9 agar plates (1.5% w/v) were prepared by pouring 20 mL of liquid LB/M9 per petri dish before drying in a laminar flow hood. Overnight cultures of both the susceptible strain and toxin-expressing strain were prepared by inoculation from glycerol stocks one day prior to the experiment. Strains were grown in a tube containing 4 mL of LB on a shaker (225 rpm) at 37°C for no longer than 12h, before the overnight culture of susceptible cells was inoculated at an OD of 0.05 in a tube containing 4 mL of LB and grown at 37°C with shaking (225 rpm). When susceptible cells reached an OD of 0.6, 200 μL of the cell suspension was added to 6 mL of melted LB/M9 agar (0.75% w/v) before pouring 6 mL of the resulting cell suspension per petri dish on the dried 20 mL of LB/M9 agar. Both the bottom agar and top agar (containing susceptible cells) of the LB/M9 agar plates contained arabinose concentrations of 0% w/v, 0.5% w/v, 1% w/v or 5% w/v. The plates were dried for 1 h. Once dried, 0.5 μL of the toxin-expressing stain was pipetted on top of the LB/M9 agar plate in serial dilutions of 10^0^ (undiluted), 10^-1^, 10^-2^, 10^-3^ and 10^-4^. The plates were dried for 15 min before placed in a static incubator at 37°C for 12 h. Plates were imaged using a gel imager and epi-fluorescent microscope.

**Propidium iodide staining**

Cell death staining was performed using propidium iodide by preparing a stock solution (1.5 mм) of propidium iodide in DMSO. Then, a working solution was prepared by diluting the stock solution with H_2_O to 150 μм. The working solution was added to the bacterial cell suspension in M9 ULGA prior to gel solidification to reach a final concentration of 5 μм.

**Determination of Relative Colony Forming Units (CFU)**

M9 ULGA gels (supplemented with 24 mм of glycerol) were formed containing E7-inducible (“Single”) or E7-inducible and S-GFP cells at a starting ratio of 1:1 and a total starting cell density of 1.6 × 10^7^ cells mL^-1^. The bacterial cells were cultured for 18 h at 37 °C in the M9 ULGA gels supplemented by varying concentrations of arabinose (0 mм – 8 mм), before re-suspending the bacterium-laden hydrogels by pipetting to form 100-fold dilutions. Then, the bacterium-containing solutions were plated onto LB agar plates containing 50 μg mL^-1^of kanamycin, which selected for S-GFP cells. The LB agar plates were than incubated for another 12 h at 37 °C, before the relative number of colony forming units arising from S-GFP cells were determined according to equation S1.

**References**

[1] D. A. Siegele, J. C. Hu, *Proc Natl Acad Sci U S A* **1997**, *94*, 8168.

[2] A. Alcinesio, O. J. Meacock, R. G. Allan, C. Monico, V. Restrepo Schild, I. Cazimoglu, M. T. Cornall, R. Krishna Kumar, H. Bayley, *Nat Commun* **2020**, *11*, 2105.

[3] E. T. Granato, K. R. Foster, *Current Biology* **2020**, *30*, 2836.

[4] D. A. I. Mavridou, D. Gonzalez, W. Kim, S. A. West, K. R. Foster, *Current Biology* **2018**, *28*, 345.

[5] E. Cascales, S. K. Buchanan, D. Duché, C. Kleanthous, R. Lloubès, K. Postle, M. Riley, S. Slatin, D. Cavard, *Microbiology and Molecular Biology Reviews* **2007**, *71*, 158.

[6] K. E. S. Avelar, L. J. F. Pinto, L. C. M. Antunes, L. A. Lobo, M. C. F. Bastos, R. M. C. P. Domingues, M. C. De Souza Ferreira, *Letters in Applied Microbiology* **1999**, *29*, 264.

[7] L. M. Guzman, D. Belin, M. J. Carson, J. Beckwith, *J Bacteriol* **1995**, *177*, 4121.

[8] C. G. Miyada, L. Stoltzfus, G. Wilcox, *Proceedings of the National Academy of Sciences* **1984**, *81*, 4120.

[9] “Benchling [Biology Software],” can be found under https://benchling.com, **2024**.

[10] L. Ghazaryan, L. Tonoyan, A. A. Ashhab, M. I. M. Soares, O. Gillor, *Arch Microbiol* **2014**, *196*, 753.
